# Value of lipocalin 2 as a potential biomarker for bacterial meningitis

**DOI:** 10.1016/j.cmi.2020.07.006

**Published:** 2021-05

**Authors:** T.T. Thanh, C. Casals-Pascual, N.T.H. Ny, N.M. Ngoc, R. Geskus, L.N.T. Nhu, N.T.T. Hong, D.T. Duc, D.D.A. Thu, P.N. Uyen, V.B. Ngoc, L.T.M. Chau, V.X. Quynh, N.H.H. Hanh, N.T.T. Thuong, L.T. Diem, B.T.B. Hanh, V.T.T. Hang, P.K.N. Oanh, R. Fischer, N.H. Phu, H.D.T. Nghia, N.V.V. Chau, N.T. Hoa, B.M. Kessler, G. Thwaites, L.V. Tan

**Affiliations:** 1) Oxford University Clinical Research Unit, Ho Chi Minh City, Viet Nam; 2) Department of Infectious Diseases, Pham Ngoc Thach University of Medicine, Ho Chi Minh City, Viet Nam; 3) Hospital for Tropical Diseases, Ho Chi Minh City, Viet Nam; 4) Department of Medicine, Vietnam National University, Ho Chi Minh City, Viet Nam; 5) Department of Clinical Microbiology, Hospital Clínic de Barcelona, CDB, Barcelona, Spain; 6) ISGlobal Barcelona, Institute for Global Health, Barcelona, Spain; 7) Target Discovery Institute, Oxford, United Kingdom; 8) Centre for Tropical Medicine and Global Health, Nuffield Department of Medicine, Oxford, United Kingdom; 9) University of Oxford, Oxford, United Kingdom

**Keywords:** Biomarkers, Central nervous system infections, Lipocalin 2, Mass spectrometry, Meningitis

## Abstract

**Objectives:**

Central nervous system (CNS) infections are common causes of morbidity and mortality worldwide. We aimed to discover protein biomarkers that could rapidly and accurately identify the likely cause of the infections, essential for clinical management and improving outcome.

**Methods:**

We applied liquid chromatography tandem mass spectrometry on 45 cerebrospinal fluid (CSF) samples from a cohort of adults with and without CNS infections to discover potential diagnostic biomarkers. We then validated the diagnostic performance of a selected biomarker candidate in an independent cohort of 364 consecutively treated adults with CNS infections admitted to a referral hospital in Vietnam.

**Results:**

In the discovery cohort, we identified lipocalin 2 (LCN2) as a potential biomarker of bacterial meningitis (BM) other than tuberculous meningitis. The analysis of the validation cohort showed that LCN2 could discriminate BM from other CNS infections (including tuberculous meningitis, cryptococcal meningitis and virus/antibody-mediated encephalitis), with sensitivity of 0.88 (95% confident interval (CI), 0.77–0.94), specificity of 0.91 (95% CI, 0.88–0.94) and diagnostic odds ratio of 73.8 (95% CI, 31.8–171.4). LCN2 outperformed other CSF markers (leukocytes, glucose, protein and lactate) commonly used in routine care worldwide. The combination of LCN2, CSF leukocytes, glucose, protein and lactate resulted in the highest diagnostic performance for BM (area under the receiver operating characteristics curve, 0.96; 95% CI, 0.93–0.99). Data are available via ProteomeXchange with identifier PXD020510.

**Conclusions:**

LCN2 is a sensitive and specific biomarker for discriminating BM from a broad spectrum of other CNS infections. A prospective study is needed to assess the diagnostic utility of LCN2 in the diagnosis and management of CNS infections.

## Introduction

Central nervous system (CNS) infections cause significant mortality and morbidity worldwide [1]. Common CNS infections include bacterial meningitis other than tuberculosis (BM), viral encephalitis, tuberculous meningitis (TBM) and cryptococcal meningitis [2,3], but there are >100 documented infectious causes of CNS infections [4]. Additionally, over the last decade, antibody-mediated causes of encephalitis (e.g. anti–*N*-methyl-d-aspartate receptor (NMDAR) encephalitis) have been recognized [5], which further challenges routine diagnostics.

Clinical features are often insufficient to discriminate the likely cause, and standard laboratory investigations identify the causative agent in <60% of cases [[6], [7], [8]]. Critically, the clinical management of CNS infections varies according to its aetiology [9]. Thus, rapid and accurate identification of the likely cause of the infection is essential to initiate appropriate therapy and improve patient outcome.

Over the last decade, mass spectrometry (MS) has emerged as a sensitive, hypothesis-free approach for the discovery of novel diagnostic biomarkers in both communicable and noncommunicable diseases [[10], [11], [12]]. Here, using a tandem MS–based approach, liquid chromatography tandem mass spectrometry (LC-MS/MS), we first searched for novel diagnostic biomarkers in cerebrospinal fluid (CSF) samples from a discovery cohort of 45 patients with brain infections. We then sought to validate our findings in an independent cohort of 364 consecutively treated adults with CNS infections.

## Materials and methods

### Setting and clinical studies

**Fig. 1:**
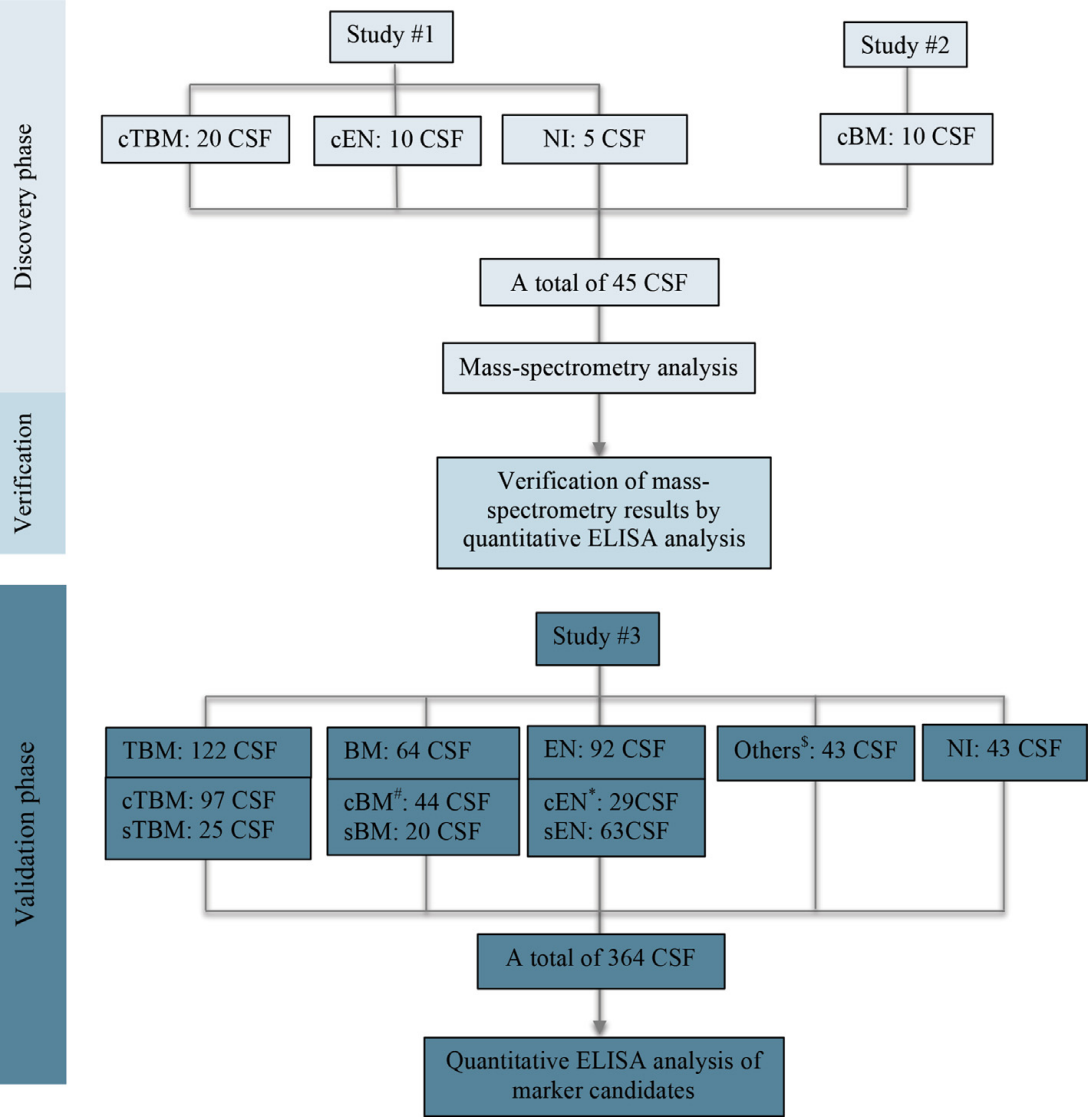
Overview of protein marker discovery phases and origin of clinical samples used for the analysis. Patients of the discovery cohort include TBM (*n* = 20), BM (*n* = 10), encephalitis (*n* = 10) and five patients with non-CNS infection. Of the ten patients with cBM, seven were infected with *Streptococcus suis* and three with *S. pneumoniae* and of the patients with encephalitis, HSV was the cause in 5, DENV in 3, JEV in 1 and MuV in 1. ^#^Including: *S. suis* (*n* = 20), *S. pneumoniae* (*n* = 6), *Escherichia coli* (*n* = 5), *Neisseria meningitidis* (*n* = 2), *Burkholderia pseudomallei* (*n* = 1), *Enterococcus faecalis* (*n* = 1), *Enterococcus gallinarum* (*n* = 1), *Streptococcus agalactiae* (*n* = 1), *Staphylococcus aureus* (*n* = 1), *Streptococcus gallolyticus* subsp. *gallolyticus* (formerly known as *S. bovis* type I) (*n* = 1) and Gram staining positive only (*n* = 5). ∗Including: HSV (*n* = 11), varicella zoster virus (*n* = 7), DENV (*n* = 5), JEV (*n* = 2), DENV/JEV (*n* = 1), MuV (*n* = 1), measles virus (*n* = 1) and influenza A virus (*n* = 1). $Including: cryptococcal meningitis (*n* = 14), anti-NMDAR encephalitis (*n* = 17), eosinophilic meningitis (*n* = 10) and neurotoxoplasmosis (*n* = 2). BM, bacterial meningitis; cBM, confirmed bacterial meningitis; cEN, confirmed encephalitis; cTBM, confirmed tuberculous meningitis; DENV, dengue virus; EN, encephalitis; HSV = herpes simplex virus; JEV, Japanese encephalitis virus; MuV, mumps virus; NI, non-CNS infections; NMDAR, *N*-methyl-d-aspartate receptor; sBM, clinically suspected bacterial meningitis; sEN, clinically suspected encephalitis; sTBM, clinically suspected tuberculous meningitis; TBM, tuberculous meningitis.

CSF samples were derived from three different clinical studies (studies 1, 2 and 3, Fig. 1 and Supplementary Materials), aiming at improving the diagnosis of adult patients with acute CNS infections. The studies were conducted in the brain infections ward of the Hospital for Tropical Diseases (HTD) in Ho Chi Minh City, Vietnam. HTD is a tertiary-care referral hospital for severe infectious diseases, including suspected CNS infections, occurring in the southern provinces of Vietnam, which has a population of over 40 million. CSF and plasma samples were collected at the time of presentation, along with demographic and clinical data and the results of routine diagnostic tests. All specimens were stored at −80°C until analysis.

### Routine diagnosis

As part of routine care, CSF specimens of patients with suspected CNS infections were cultured and/or examined by microscopy for the detection of bacteria (including *Mycobacterium tuberculosis*) and fungi with the use of standard methods (Supplementary Table S1) [13]. Herpes simplex virus (HSV) PCR was performed on CSF from those with suspected viral encephalitis. Varicella zoster virus (VZV) PCR and serologic testing for IgM against dengue virus (DENV) IgM, Japanese encephalitis virus (JEV) or mumps virus (MuV) was performed if clinically indicated and when testing for other pathogens was negative. Diagnosis of measles was based on compatible clinical features and the presence of measles IgM.

### Assignment of CNS infection diagnosis

Assignment of the CNS infection cause (TBM, BM, cryptococcal meningitis, eosinophilic meningitis or anti-NMDAR encephalitis) was first based on the results of standard laboratory investigations. The diagnosis was confirmed if the relevant infectious agent was identified in the CSF by microbial investigation (e.g. some combination of routine culture, Gram stain and PCR in case of BM) (Supplementary Tables S1 and S2). Otherwise, patients were considered as having clinically suspected CNS infections (TBM, BM, encephalitis) on the basis of treatment response and/or the clinical judgement of the treating physicians. Because of the focus of the present study, probable and possible TBM (defined by the Marais criteria [14]) were regarded as clinically suspected TBM. CNS infection was excluded in patients who had no meningeal signs, CSF laboratory parameters in normal ranges and negative results of microbiologic and serologic investigations.

### Sample preparation and LC-MS/MS analysis

CSF was analysed as individual samples using proteomic platforms available at the Target Discovery Institute, University of Oxford. Proteomic analysis was carried out as described in the Supplementary Materials. In brief, MS/MS spectra were searched using the Central Proteomics Facilities Pipeline in a meta-search against the UniProt Homo Sapiens Reference proteome (retrieved October 15, 2014), allowing for a precursor mass tolerance of 10 ppm and a fragment ion tolerance of 0.05 Da. Deamidation on asparagine and glutamine and oxidation on methionine were included as variable modifications. The peptide false-discovery rate was set at 1%. Separation of CNS infection diagnostic groups based on the obtained peptide/protein profiles was performed by Perseus 1.6.6.0 software [15].

### Measurement of LCN2 concentration by quantitative enzyme-linked immunosorbent assay

Measurement of lipocalin 2 (LCN2) concentration was performed on CSF samples and a subset of plasma samples of the study participants using monoclonal antibody–based Quantikine enzyme-linked immunosorbent assay (ELISA) kits (R&D Systems, Minneapolis, MN, US). The experiments were performed according to the manufacturer's instruction. (The costs is about US$10 per test.)

### Statistical analysis

Continuous variables were compared by the Mann-Whitney *U* test, or the Kruskal-Wallis test or Wilcoxon signed-rank test. The correlation between continuous variables was assessed by the Spearman correlation test. All statistical tests were two sided. The area under the receiver operating characteristic curve (AUROC) was used to quantify the diagnostic performance of biomarkers for a given diagnosis. The cutoff values for outcome prediction were selected on the basis of the highest sum of sensitivity and specificity. A logistic regression model was used to evaluate the diagnostic performance of two or more variables combined. All continuous variables were modeled as linear terms.

All analyses were performed by SPSS 23.0 (IBM, White Plains, NY, USA), and all figures were generated by GraphPad Prism 5.04 (GraphPad Software, La Jolla, CA, USA).

### Ethics

The study was approved by institutional review board of HTD and the Oxford Tropical research ethics committee (OxTREC). Written informed consent was obtained from each participant, or a relative if the patient was incapacitated.

## Results

### Baseline characteristics of study population

#### Discovery cohort

We selected a total of 45 patients for the discovery phase: 40 patients with laboratory confirmed CNS infections (TBM, BM and encephalitis) and five patients with non-CNS infection (Fig. 1). The cohort's clinical characteristics and outcomes are presented in Supplementary Table S3.

#### Validation cohort

We enrolled 364 consecutive adult patients with suspected CNS infection into the validation study (Fig. 1). The baseline characteristics, clinical outcomes and results of aetiologic investigations of the cohort are presented in Supplementary Table S3 and Fig. 1. After the exclusion of 43 patients without CNS infections (Supplementary Table S4), the causes were confirmed in 64% (207/321) of the patients with CNS infections. TBM was the most frequent diagnosis, followed by viral encephalitis and BM. The remaining patients included those with anti-NMDAR encephalitis, cryptococcal meningitis, parasitic eosinophilic meningitis and neurotoxoplasmosis (Fig. 1). Of the patients with TBM, BM and viral encephalitis, a confirmed diagnosis was established in 80% (97/122), 69% (44/64) and 32% (29/92), respectively. Specific causes of BM and encephalitis are presented in Fig. 1.

### Biomarker discovery

**Fig. 2:**
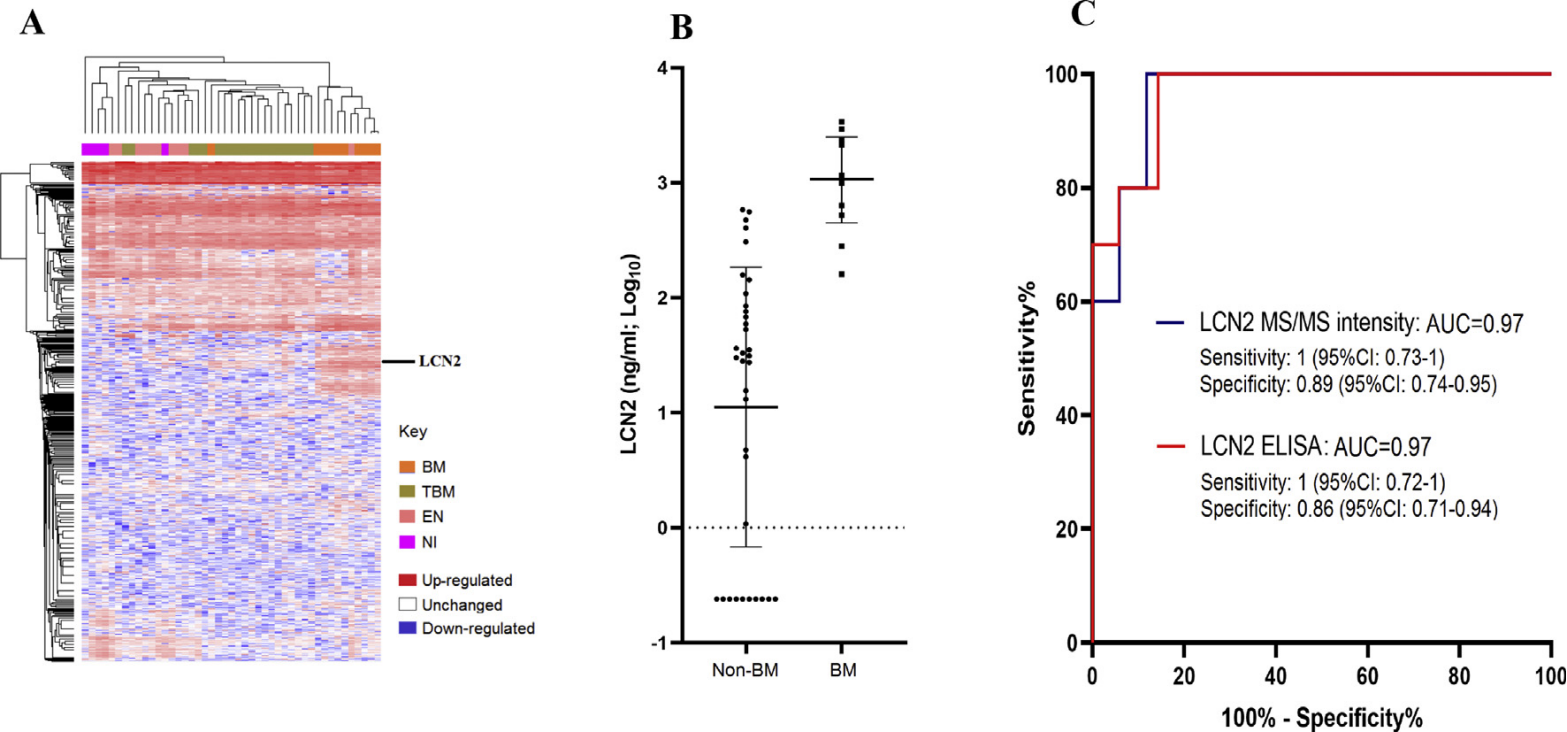
Results of LC-MS/MS and LCN2 ELISA analysis of discovery cohort. (A) Heat map showing clinical entities clustering based on protein/peptide profiles obtained from label-free quantitative MS analysis of 45 patients of the discovery phase. Columns represent clinical entities; rows, individual proteins; and black line, relative position of LCN2. (B) Dot plots demonstrating the difference in CSF LCN2 levels between BM and non-BM groups obtained from quantitative ELISA analysis, (C) AUROC based on LCN2 levels measured by MS (blue line) and quantitative ELISA (red line) analysis. Colors indicate differentially expressed proteins: red indicates upregulated; white, unchanged; and blue, downregulated. AUROC, area under the receiver operating characteristics curve; BM, bacterial meningitis; CSF, cerebrospinal fluid; ELISA, enzyme-linked immunosorbent assay; LC-MS/MS, liquid chromatography tandem mass spectrometry; LCN2, lipocalin 2; MS, mass spectrometry; non-BM, nonbacterial meningitis (encephalitis, tuberculous meningitis or non–central nervous system infections).

LC-MS/MS analysis of 45 CSF samples of the discovery cohort identified a total of 1012 proteins. Subsequent analysis identified a total of 729 quantifiable protein signatures that were clinical-entity specific, especially for patients with BM (Fig. 2(A)). Of these, 60 and 19 were significantly expressed in the CSF of patients with BM and TBM, respectively (Supplementary Table S5 and Supplementary Data). No diagnostic biomarker candidate was found in patients with viral encephalitis.

Of the protein candidates identified in the BM group, lipocalin 2 (LCN2), which is also known as neutrophil gelatinase-associated lipocalin (NGAL) had a sensitivity of 1 (95% confidence interval (CI), 0.73–1) and a specificity of 0.89 (95% CI, 0.74–0.95) for prediction of BM (AUROC, 0.97; 95% CI, 0.9–1). It was thus selected for further evaluation.

### LCN2 ELISA analysis to verify results of original LC-MS/MS

To verify the LC-MS/MS findings, we performed quantitative ELISA analysis of the 45 CSF samples used for the discovery phase. Subsequently, the result suggested LCN2 concentration of ≥59 ng/mL could accurately distinguish BM from TBM, encephalitis and non-CNS infections groups, with an AUROC value of 0.97 (95% CI, 0.92–1), corresponding to a sensitivity of 1 (95% CI, 0.72–1) and a specificity of 0.86 (95% CI, 0.71–0.94) (Fig. 2(B) and (C)). Thus, the diagnostic values of LCN2 based on the results of quantitative ELISA analysis confirmed the original finding of the LC-MS/MS analysis.

### CSF LCN2 concentrations in validation cohort

**Fig. 3:**
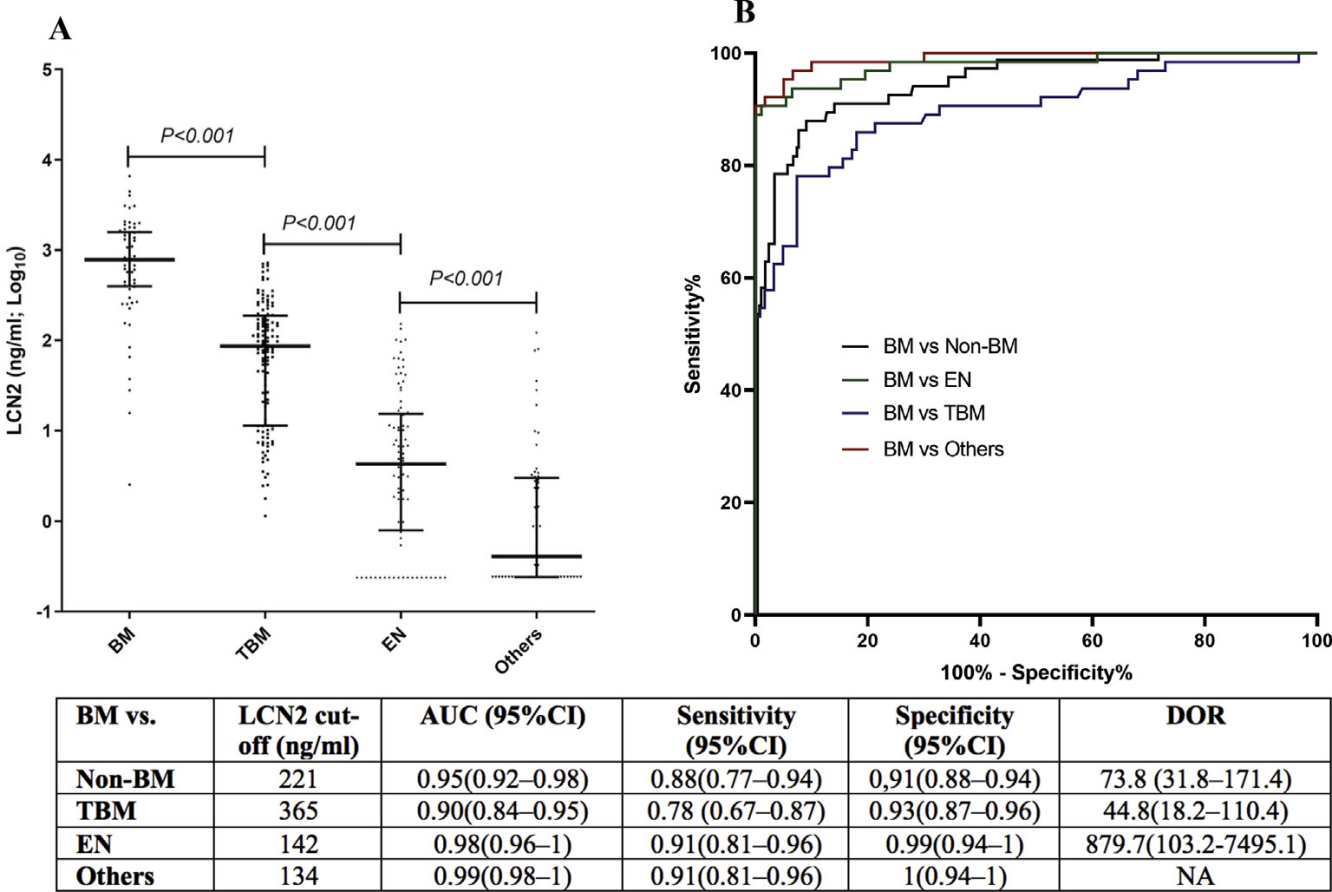
Results of LCN2 ELISA and AUROC analysis of validation cohort. (A) LCN2 concentrations in patients with meningitis, tuberculous meningitis, encephalitis and others (cryptococcal meningitis, anti-NMDAR encephalitis, eosinophilic meningitis, neurotoxoplasmosis and non-CNS infections). (B) AUROC showing diagnostic values of LCN2 in discriminating bacterial meningitis from other CNS infection entities. Others indicates patients with other CNS infections (cryptococcal meningitis, anti-NMDAR encephalitis, neurotoxoplasmosis or eosinophilic meningitis) or non-CNS infections. AUROC, area under the receiver operating characteristics curve; BM, bacterial meningitis; CNS, central nervous system; CSF, cerebrospinal fluid; ELISA, enzyme-linked immunosorbent assay; LCN2, lipocalin 2; NMDAR, *N*-methyl-d-aspartate receptor; non-BM, nonbacterial meningitis.

LCN2 concentrations were significantly different among the CNS infection groups, with the highest concentration observed in the BM group (median, 778. 8 ng/mL; range, 2.5–6566.3 ng/mL), followed by TBM groups (median, 86.3 ng/mL; range, 1.1–723.4 ng/mL) (Fig. 3(A)). In contrast, LCN2 was almost absent or detected at very low levels in CSF of patients with anti-NMDAR encephalitis (median, 0.9 ng/mL; range, 0.2–27.8 ng/mL) or without CNS infection (median, 0.2 ng/mL; range, 0.2–120.3 ng/mL) (Fig. S1(A)). Of the patients with BM, CSF LCN2 levels were comparable between major pathogen groups (Fig. S1(B)) but higher in those with a confirmed diagnosis than in those without a bacterium identified (Fig. S1(A)), while the duration of illness at enrolment was similar between the two groups (data not shown). Illness duration, however, was negatively correlated with CSF LCN2 levels of BM patients but positively correlated that of TBM patients (Fig. S2). Finally, LCN2 levels were positively associated with poor in-hospital outcomes of TBM patients, but there was no association between LCN2 levels and in-hospital outcome in the remaining patient groups (Fig. S3).

### Diagnostic performance of CSF LCN2

Analysis of LCN2 concentrations obtained from the validation cohort demonstrated that LCN2 could accurately discriminate BM from other CNS infections, with AUROC ranging from 0.9 (for BM vs. TBM, LCN2 concentration cutoff of 365 ng/mL) to 0.99 (BM vs. other CNS infections (i.e. non-encephalitis or non-TBM), LCN2 concentration cutoff: 134 ng/mL) and a diagnostic odd ratio of 44.8 or above (Fig. 3(B)).

**Fig. 4:**
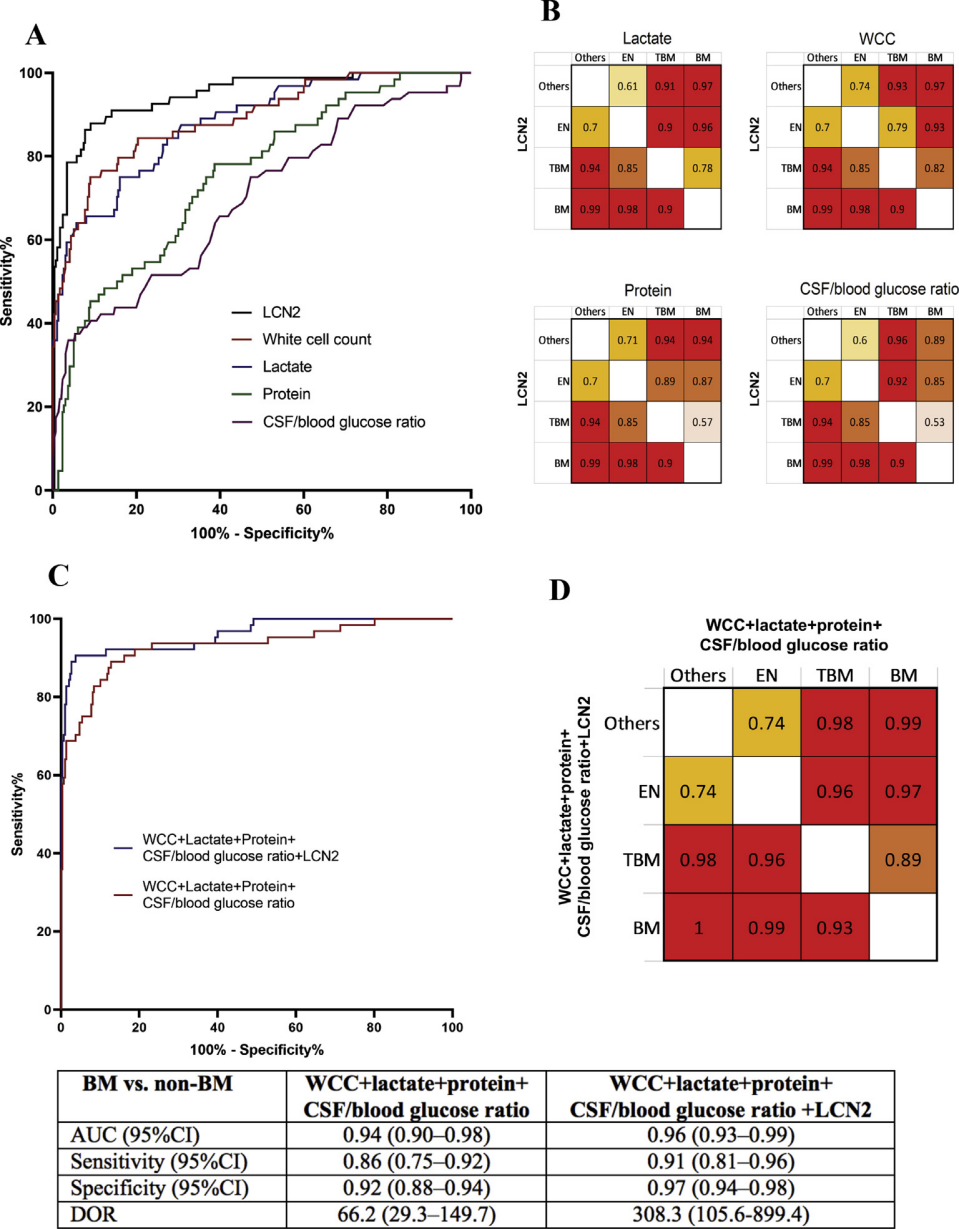
Diagnostic values of LCN2 in predicting bacterial meningitis in comparison and in combination with existing CSF parameters. (A) AUROC showing that LCN2 is better than the existing CSF parameters in distinguishing between bacterial meningitis with other CNS infections (tuberculous meningitis, encephalitis, anti-NMDAR encephalitis, cryptococcal meningitis, neurotoxoplasmosis, eosinophilic meningitis or non-CNS infections). (B) AUROC values of subgroup analyses. (C) AUROC showing that LCN2 significantly improves the discriminatory ability of the diagnostic model for bacterial meningitis using the remaining CNS infections groups as controls. (D) AUROC values of subgroup analyses. AUROC, area under the receiver operating characteristics curve; CNS, central nervous system; CSF, cerebrospinal fluid; LCN2, lipocalin 2; NMDAR, *N*-methyl-d-aspartate receptor; WCC, white blood cell (leukocyte) count.

LCN2 outperformed the existing biomarkers (leukocytes, protein, lactate and glucose) in discriminating between BM and other CNS infections (including TBM and encephalitis) (Fig. 4(A) and (B)). When LCN2 was combined with leukocytes, protein, lactate and glucose concentrations in CSF, the diagnostic model consisting of LCN2 and these four CSF parameters provided the highest discriminatory ability for BM (Fig. 4(C)). In terms of discriminating between BM and all other CNS infections, the predictive values for BM based on AUROC and the diagnostic odd ratio increased from 0.94 (95% CI, 0.80–0.98) to 0.96 (95% CI, 0.93–0.99), and 66.2 to 308.3 when LCN2 was added to the CSF parameter–based model (Fig. 4(C)). Subgroup analyses showed that adding LCN2 to the diagnostic model consisting of leukocytes, protein, lactate and glucose improved the AUROC for differentiation between BM and TBM from 0.89 to 0.93, but slightly improved the discrimination between BM from other infections such as encephalitis and patients with other CNS infections (Fig. 4(D) and Fig. S4). LCN2 did not, however, help distinguish confirmed from suspected BM (Supplementary Table S6).

### Association between CSF and plasma concentrations of LCN2

We assessed if plasma LCN2 can be a surrogate of CSF LCN2 in a subset of 22 patients with BM (14 laboratory confirmed, 8 clinically suspected). Plasma LCN2 concentration was, however, significantly lower than that of CSF, with median (range) values of 147.9 (33.7–194.8) ng/mL vs. CSF LCN2, at 472.1 (15.7–3102.3) ng/mL (p < 0.001). There was no correlation between CSF and plasma LCN2 (Spearman R, 0.37; p 0.08), suggesting that LCN2 is intrathecally produced in response to the bacterial invasion of the CNS.

## Discussion

Here, using a MS-based approach, we initially identified LCN2 as a potential diagnostic marker for BM. Additional validation work on an independent cohort showed that LCN2 could accurately discriminate BM from other CNS infections. LCN2 also outperformed existing BM diagnostic makers (CSF leukocytes, protein, glucose and lactate) that are currently used as part of routine care. A diagnostic model consisting of LCN2 and these four CSF parameters gave the best diagnostic performance for BM. Our data thus suggest that LCN2 can act as an independent diagnostic maker of BM alone or in combination with other CSF parameters.

LCN2 is secreted by neutrophils, hepatocytes and renal tubular cells [16]. It has antibacterial properties, inhibiting bacterial growth via the interference of bacterial iron uptake [16]. LCN2 has recently been recognized as a sensitive biomarker for the diagnosis of severe bloodstream infection [17] and pneumonia caused by *Streptococcus pneumoniae* [18]. High concentrations of LCN2 in the CSF of patients with BM have been previously reported [19,20]. However, previous studies only focused on quantifying LCN2 concentrations in patients with confirmed BM and viral encephalitis, and did not compare the performance of LCN2 against commonly used CSF markers such as leukocytes, glucose, protein and lactate. Our study was conducted in Vietnam and included patients with many different CNS infections, including bacterial, fungal, tuberculous, viral and parasitic meningitis, as well as anti-NMDAR encephalitis. Additionally, we also compared the diagnostic performance of LCN2 against that of CSF markers commonly used as part of routine care worldwide. As such, our results have expanded our knowledge about the relation between LCN2 and CNS infections and provide strong evidence that LCN2 is a highly sensitive biomarker for discriminating BM from a broad spectrum of other CNS infections.

The differences in CSF LCN2 levels between laboratory-confirmed and clinically suspected BM groups pointed to the association between the host responses and an ongoing infection (i.e. the presence of a bacterial pathogen in clinical samples at the time of collection). This is in agreement with previous studies showing that the decrease of plasma LCN2 level was correlated with the success of antibiotic treatment in patients with bacteraemia [21]. Collectively, CSF LCN2 might also be a useful marker for treatment response assessment. Therefore, further research should aim at defining the optimal cutoff of LCN2 concentrations that can be used to inform the administration or withdrawal of antibiotics in patients with BM.

While the current high cost of LC-MS/MS remains a major barrier preventing it from being used for clinical research in resource-constrained settings, our study has some other limitations. We did not explore the utility potential of other biomarker candidates identified in the discovery cohort (e.g. CSF cathelicidin for BM [22]) detected by original LC-MS/MS analysis. Likewise, we did not assess the diagnostic performance of LCN2 against and/or in combination with newly proposed biomarkers for CNS infections such as procalcitonin. interleukin 6 [23], persephin [24] or heparin-binding protein for BM [10,12,[25], [26], [27]] and CSF lipoarabinomannan for TBM [28]. Additionally, we only focused our analysis on adults, leaving the utility potential of LCN2 in paediatric CNS infections unknown. Finally, the small sample size (*n* = 45) of the discovery cohort may have lowered the power to detect other potential candidate markers.

In spite of these limitations, the strengths of our study include that it represents the largest and most comprehensive MS-based biomarker discovery investigation focusing on patients with various clinical entities of CNS infections to date [10]. Our study includes all the major infectious causes of CNS infections seen globally. Additionally, because our study was conducted at a single major tertiary-care referral hospital, all routine diagnostic approaches and patient assessments were consistent over the course of the study, thereby minimizing potential bias.

To summarize, our study showed for the first time that LCN2 is a highly sensitive biomarker for accurate prediction of BM in adults, especially when used alongside other standard CSF parameters. Prospective studies are needed to assess the utility potential of LCN2 in the diagnosis and management of CNS infections, and whether it can be used in settings with limited laboratory capacity to improve outcomes from these devastating conditions.

## Transparency declaration

Funded by the 10.13039/100010269Wellcome Trust of Great Britain (106680/B/14/Z and 204904/Z/16/Z awarded to GT and LVT, respectively). All authors report no conflicts of interest relevant to this article.

## References

[1] Roth G.A., Abate D., Abate K.H., Abay S.M., Abbafati C., Abbasi N. (2018). Global, regional, and national age-sex–specific mortality for 282 causes of death in 195 countries and territories, 1980–2017: a systematic analysis for the Global Burden of Disease Study 2017. Lancet.

[2] Kanjilal S., Cho T.A., Piantadosi A. (2019). Diagnostic testing in central nervous system infection. Semin Neurol.

[3] Bloch K.C., Glaser C.A. (2015). Encephalitis surveillance through the emerging infections program, 1997–2010. Emerg Infect Dis.

[4] Dubot-Peres A., Mayxay M., Phetsouvanh R., Lee S.J., Rattanavong S., Vongsouvath M. (2019). Management of central nervous system infections, Vientiane, Laos, 2003–2011. Emerg Infect Dis.

[5] Dalmau J., Graus F. (2018). Antibody-mediated encephalitis. N Engl J Med.

[6] Granerod J., Ambrose H.E., Davies N.W.S., Clewley J.P., Walsh A.L., Morgan D. (2010). Causes of encephalitis and differences in their clinical presentations in England: a multicentre, population-based prospective study. Lancet Infect Dis.

[7] Glaser C.A., Honarmand S., Anderson L.J., Schnurr D.P., Forghani B., Cossen C.K. (2006). Beyond viruses: clinical profiles and etiologies associated with encephalitis. Clin Infect Dis.

[8] Roux A., Houcke S., Sanna A., Mathien C., Mayence C., Gueneau R. (2019). Clinical features, diagnosis, and outcome of encephalitis in French Guiana. Am J Trop Med Hyg.

[9] van Ettekoven C.N., van de Beek D., Brouwer M.C. (2017). Update on community-acquired bacterial meningitis: guidance and challenges. Clin Microbiol Infect.

[10] Bharucha T., Gangadharan B., Kumar A., de Lamballerie X., Newton P.N., Winterberg M. (2019). Mass spectrometry–based proteomic techniques to identify cerebrospinal fluid biomarkers for diagnosing suspected central nervous system infections. A systematic review. J Infect.

[11] Geyer P.E., Holdt L.M., Teupser D., Mann M. (2017). Revisiting biomarker discovery by plasma proteomics. Mol Syst Biol.

[12] Njunge J.M., Oyaro I.N., Kibinge N.K., Rono M.K., Kariuki S.M., Newton C.R. (2017). Cerebrospinal fluid markers to distinguish bacterial meningitis from cerebral malaria in children. Wellcome Open Res.

[13] Tan le V., Thai le H., Phu N.H., Nghia H.D., Chuong L.V., Sinh D.X. (2014). Viral aetiology of central nervous system infections in adults admitted to a tertiary referral hospital in southern Vietnam over 12 years. PLoS Negl Trop Dis.

[14] Marais S., Thwaites G., Schoeman J.F., Torok M.E., Misra U.K., Prasad K. (2010). Tuberculous meningitis: a uniform case definition for use in clinical research. Lancet Infect Dis.

[15] Tyanova S., Temu T., Sinitcyn P., Carlson A., Hein M.Y., Geiger T. (2016). The Perseus computational platform for comprehensive analysis of (prote)omics data. Nat Methods.

[16] Schmidt-Ott K.M., Mori K., Li J.Y., Kalandadze A., Cohen D.J., Devarajan P. (2007). Dual action of neutrophil gelatinase-associated lipocalin. J Am Soc Nephrol.

[17] Herberg J., Huang H., Thezenas M.L., Janes V., Carter M., Gormley S. (2019). Lipocalin-2 is a sensitive and specific marker of bacterial infection in children. bioRxiv.

[18] Huang H., Ideh R.C., Gitau E., Thezenas M.L., Jallow M., Ebruke B. (2014). Discovery and validation of biomarkers to guide clinical management of pneumonia in African children. Clin Infect Dis.

[19] Guiddir T., Deghmane A.E., Giorgini D., Taha M.K. (2014). Lipocalin 2 in cerebrospinal fluid as a marker of acute bacterial meningitis. BMC Infect Dis.

[20] Lippi G., Avanzini P., Calzetti C., Caleffi A., Pipitone S., Musa R. (2014). The role of neutrophil gelatinase-associated lipocalin (NGAL) in cerebrospinal fluids for screening of acute bacterial meningitis. Clin Lab.

[21] Fjaertoft G., Foucard T., Xu S., Venge P. (2005). Human neutrophil lipocalin (HNL) as a diagnostic tool in children with acute infections: a study of the kinetics. Acta Paediatr.

[22] Savonius O., Helve O., Roine I., Andersson S., Saukkoriipi A., Gonzalez Mata A. (2018). Cerebrospinal fluid cathelicidin correlates with the bacterial load and outcomes in childhood bacterial meningitis. Pediatr Infect Dis J.

[23] Takahashi W., Nakada T.A., Abe R., Tanaka K., Matsumura Y., Oda S. (2014). Usefulness of interleukin 6 levels in the cerebrospinal fluid for the diagnosis of bacterial meningitis. J Crit Care.

[24] Stubljar D., Kopitar A.N., Groselj-Grenc M., Suhadolc K., Fabjan T., Skvarc M. (2015). Diagnostic accuracy of presepsin (sCD14-ST) for prediction of bacterial infection in cerebrospinal fluid samples from children with suspected bacterial meningitis or ventriculitis. J Clin Microbiol.

[25] Reshi Z., Nazir M., Wani W., Malik M., Iqbal J., Wajid S. (2017). Cerebrospinal fluid procalcitonin as a biomarker of bacterial meningitis in neonates. J Perinatol.

[26] Li W., Sun X., Yuan F., Gao Q., Ma Y., Jiang Y. (2017). Diagnostic accuracy of cerebrospinal fluid procalcitonin in bacterial meningitis patients with empiric antibiotic pretreatment. J Clin Microbiol.

[27] Linder A., Akesson P., Brink M., Studahl M., Bjorck L., Christensson B. (2011). Heparin-binding protein: a diagnostic marker of acute bacterial meningitis. Crit Care Med.

[28] Siddiqi O.K., Birbeck G.L., Ghebremichael M., Mubanga E., Love S., Buback C. (2019). Prospective cohort study on performance of cerebrospinal fluid (CSF) Xpert MTB/RIF, CSF lipoarabinomannan (LAM) lateral flow assay (LFA), and urine LAM LFA for diagnosis of tuberculous meningitis in Zambia. J Clin Microbiol.

