# Supplementary material for: Value of lipocalin 2 as a potential biomarker for bacterial meningitis
Source: Clin Microbiol Infect. 2021 May;27(5):724–30. doi: 10.1016/j.cmi.2020.07.006 (PMC8128987; doi:10.1016/j.cmi.2020.07.006)
Supplement: Multimedia component 2 [file mmc2.docx]

**SUPPLEMENTARY MATERIALS FOR**

**Value of Lipocalin 2 as a potential biomarker for bacterial meningitis**

Tran Tan Thanh^1†^, Climent Casals-Pascual^2†^, Nguyen Thi Han Ny^1^, Nghiem My Ngoc, Ronald Geskus^1^,Le Nguyen Truc Nhu^1^, Nguyen Thi Thu Hong^1^, Du Trong Duc^3^, Do Dang Anh Thu^1^, Phan Nha Uyen^1,#^, Vuong Bao Ngoc^1,**^, Le Thi My Chau^4^, Van Xuan Quynh^4^, Nguyen Ho Hong Hanh^5^, Nguyen Thuy Thuong Thuong^1^, Le Thi Diem^5^, Bui Thi Bich Hanh^3^, Vu Thi Ty Hang^1^, Pham Kieu Nguyet Oanh^4^, Roman Fischer^6,7^, Nguyen Hoan Phu^1,5^, Ho Dang Trung Nghia^1,3,4^, Nguyen Van Vinh Chau^4^, Ngo Thi Hoa^1^, Benedikt M. Kessler^6,7^, Guy Thwaites^1,8^, and Le Van Tan^1^*

**Affiliations:**

^1^Oxford University Clinical Research Unit, Ho Chi Minh City, Vietnam.

^2^Department of Clinical Microbiology, Hospital Clínic de Barcelona, CDB; ISGlobal, Barcelona, Institute for Global health, Barcelona, Spain

^3^Department of infectious diseases, Pham Ngoc Thach University of Medicine, Ho Chi Minh City, Vietnam

^4^Hospital for Tropical Diseases, Ho Chi Minh City, Vietnam

^5^Department of Medicine, Vietnam National University, Ho Chi Minh City, Vietnam

^6^Target Discovery Institute, Nuffield Department of Medicine, University of Oxford, Oxford, United Kingdom

^7^University of Oxford, Oxford, United Kingdom

^8^Centre for Tropical Medicine and Global Health, Nuffield Department of Medicine, University of Oxford, Oxford, UK

Current affiliations: ^#^Duy Tan University, Da Nang, Vietnam, **Taleed Academy, Ho Chi Minh City, Vietnam

*To whom correspondence should be addressed: tanlv@oucru.org

^†^Authors contributed equally to this work.

**Word counts:** Abstract: 250, text: 2500

**Keywords:** Meningitis, central nervous system infections, mass-spectrometry, lipocalin 2, biomarkers

**Running title:** Lipocalin 2: a potential biomarker for bacterial meningitis

**The clinical studies**

The clinical study #1 entitled “expanding the laboratory diagnosis of tuberculous meningitis and meningoencephalitis in Vietnam” was conducted during January 2015–September 2016 [1]. As per the study protocol, any adult (≥18 years) with a suspected CNS infection and requirement for lumbar puncture was eligible for enrolment. Patients were excluded if pyogenic bacterial meningitis (very cloudy or pus-like CSF) was suspected, lumbar puncture was contra-indicated (Patient was excluded if lumbar puncture was contra-indicated (e.g. in case there is a risk for cerebral herniation (including space-occupying lesion with mass effect), a risk of bleeding (thrombocytopenia, coagulopathies and anticoagulant drugs) or a local infection at the puncture site), or no informed consent was obtained.

The clinical study #2 focused on the immunological responses in bacterial meningitis patients, especially those infected with *Streptococcus suis*, and was conducted during 2015 and 2017. Any patient (≥16 years) with suspected pyogenic bacterial meningitis (very cloudy or pus-like CSF) was eligible for enrolment. Patient was excluded if lumbar puncture was contra-indicated, or no informed consent was obtained.

The clinical study #3 started in September 2017 and is on-going. The study aims to explore the potential diagnostic utility of next-generation sequencing and mass-spectrometry in CNS infections. Any patient (≥16 years) with suspected CNS infection and requirement for lumbar puncture was eligible for enrolment. Patients were excluded if no written informed consent was obtained.

**Details of proteomics procedure**

Protein extraction, precipitation and in-solution digestion

Samples of CSF were thawed on ice and digested using heat-

stable immobilized trypsin (SMART digest; Thermo Fisher Sci-

entific, Loughborough, UK). CSF (50ll) was mixed with 150ll

of SMART digest buffer and added to SMART digest plates.

Samples were incubated at 708C with shaking at 1,400rpm for

60 minutes. Digested samples were desalted using SOLAl

plates and dried by vacuum centrifugation. Samples were resus-

pended in 20ll buffer A (2% acetonitrile, 0.1% formic acid in

water) and kept at –208C until analysis. Peptide concentrations

were assayed using a Pierce (Thermo Fisher Scientific, UK)

quantitative colorimetric peptide assay according to the manu-

facturer’s instructions. A pooled sample was produced by com-

bining equal quantities of digested peptide from each individual

sample and injected after every 10th sample for use in quality-

control analysis.

Samples of CSF were thawed on ice and digested using heat-

stable immobilized trypsin (SMART digest; Thermo Fisher Sci-

entific, Loughborough, UK). CSF (50ll) was mixed with 150ll

of SMART digest buffer and added to SMART digest plates.

Samples were incubated at 708C with shaking at 1,400rpm for

60 minutes. Digested samples were desalted using SOLAl

plates and dried by vacuum centrifugation. Samples were resus-

pended in 20ll buffer A (2% acetonitrile, 0.1% formic acid in

water) and kept at –208C until analysis. Peptide concentrations

were assayed using a Pierce (Thermo Fisher Scientific, UK)

quantitative colorimetric peptide assay according to the manu-

facturer’s instructions. A pooled sample was produced by com-

bining equal quantities of digested peptide from each individual

sample and injected after every 10th sample for use in quality-

control analysis.

Samples of CSF were thawed on ice and digested using heat-

stable immobilized trypsin (SMART digest; Thermo Fisher Sci-

entific, Loughborough, UK). CSF (50ll) was mixed with 150ll

of SMART digest buffer and added to SMART digest plates.

Samples were incubated at 708C with shaking at 1,400rpm for

60 minutes. Digested samples were desalted using SOLAl

plates and dried by vacuum centrifugation. Samples were resus-

pended in 20ll buffer A (2% acetonitrile, 0.1% formic acid in

water) and kept at –208C until analysis. Peptide concentrations

were assayed using a Pierce (Thermo Fisher Scientific, UK)

quantitative colorimetric peptide assay according to the manu-

facturer’s instructions. A pooled sample was produced by com-

bining equal quantities of digested peptide from each individual

sample and injected after every 10th sample for use in quality-

control analysis.

CSF sample preparation was performed essentially as described [2]. In brief, CSF liquid material per patient was thawed on ice and digested using heatstable immobilized trypsin (SMART digest; Thermo Fisher Scientific). CSF (50 μl) was mixed with 150 μl of SMART digest buffer and added to SMART digest plates. Samples were protelysed by incubation at 70 ^o^C and shaking at 1,400 rpm for 60 minutes. Digested samples were desalted using SOLA plates (ThermoFisher Scientific), dried by vacuum centrifugation and resuspended in 20 μl buffer A (2 % acetonitrile, 0.1 % formic acid in water). Samples were kept at -20 ^o^C until analysis. Peptide concentrations were assayed using a Pierce (ThermoFisher Scientific) quantitative colorimetric peptide assay according to the manufacturer’s instructions. A pooled sample was produced by combining equal quantities of digested peptide from each individual sample and injected after every 10th sample for use in quality control (QC) analysis.

Mass spectrometry analysis

Proteomics analysis of peptides was performed essentially as described [2-4]. In brief, peptide samples prepared as described above were analysed by nano ultra-high performance liquid chromatography tandem mass spectrometry (nUHPLC LC-MS/ MS) using a Dionex Ultimate 3000 UHPLC (Thermo Fisher Scientific) coupled to a Q Exactive HF tandem mass spectrometer (Thermo Fisher Scientific). 500 nL peptide material from each sample was injected and analyzed using a 60-minute linear gradient at a flow rate of ~250-nl/minute. The gradient used to elute the peptides was 3 minutes with 2% buffer B (0.1 % trifluoroacetic acid and 5 % dimethyl sulfoxide in acetonitrile) / 98 % buffer A (2 % acetonitrile, 0.1 % formic acid in water), increasing to 5 % B by 6 minutes, followed by a linear ramp up to 35 % B at 63 minutes. Data were acquired with a resolution of 60,000 full width at half maximum ion intensity with a mass/charge ratio of 400 and a lock mass of 445.120025 m/z. The 12 most abundant precursor ions in each MS1 scan were selected for fragmentation by high energy collisional dissociation at a normalized collision energy of 28 followed by peak exclusion for 27 seconds. Raw MS data were analysed using MaxQuant software (v1.6.0.1). MS/MS spectra were searched against the UniProt Homo Sapiens Reference proteome (retrieved January 6, 2017), allowing for a precursor mass tolerance of 10 ppm and a fragment ion tolerance of 0.05 Da. Deamidation on asparagine and glutamine and oxidation on methionine were included as variable modifications. The peptide false discovery rate (FDR) was set at 1 %. Separation of CNS infection diagnostic groups based on the obtained peptide/protein profiles was performed using Perseus software version 1.6.6.0 [5].

**References**

1. Heemskerk, A.D., J. Donovan, D.D.A. Thu, S. Marais, L. Chaidir, V.T.M. Dung, et al., *Improving the microbiological diagnosis of tuberculous meningitis: A prospective, international, multicentre comparison of conventional and modified Ziehl-Neelsen stain, GeneXpert, and culture of cerebrospinal fluid.* J Infect, 2018. **77**: p. 509-515.

2. Thompson, A.G., E. Gray, M.L. Thezenas, P.D. Charles, S. Evetts, M.T. Hu, et al., *Cerebrospinal fluid macrophage biomarkers in amyotrophic lateral sclerosis.* Ann Neurol, 2018. **83**: p. 258-268.

3. Fischer, R. and B.M. Kessler, *Gel-aided sample preparation (GASP)--a simplified method for gel-assisted proteomic sample generation from protein extracts and intact cells.* Proteomics, 2015. **15**: p. 1224-9.

4. Fye, H.K.S., P. Mrosso, L. Bruce, M.L. Thezenas, S. Davis, R. Fischer, et al., *A robust mass spectrometry method for rapid profiling of erythrocyte ghost membrane proteomes.* Clin Proteomics, 2018. **15**: p. 14.

5. Tyanova, S., T. Temu, P. Sinitcyn, A. Carlson, M.Y. Hein, T. Geiger, et al., *The Perseus computational platform for comprehensive analysis of (prote)omics data.* Nat Methods, 2016. 13(9): p. 731-40.

**Table S1. Diagnostic tests carried out as part of routine care and/or as per the study protocols**

|  | **Study #1** | **Study #2** | **Study #3** |
| --- | --- | --- | --- |
| Gram stain | Y | Y | Y |
| India Ink stain |  |  |  |
| Bacterial culture | Y | Y | Y |
| Ziehl-Neelsen staining | Y | Y | Y |
| GenXpert | Y | Y | Y |
| MGIT | Y | Y | Y |
| *S. suis* PCR | Y | Y | Y |
| *S. pneumoniae* PCR | Y | Y | Y |
| *N. meningitidis* PCR | Y | Y | Y |
| 16S rRNA PCR | ND | Y | Y |
| HSV PCR | Y | Y | Y |
| VZV PCR | Y | Y | Y |
| DENV PCR | ND | ND | Y |
| JEV PCR | ND | ND | Y |
| Flavivirus PCR | ND | ND | Y |
| Enterovirus PCR | ND | ND | Y |
| Influenza A virus | Y | Y | Y |
| Mumps virus PCR | ND | ND | Y |
| Zika virus PCR | ND | ND | Y |
| *Angiostrongylus cantonensis* PCR | ND | ND | Y |
| Cryptococcal FLA | Y | Y | Y |
| DENV serology | Y | Y | Y |
| JEV serology | Y | Y | Y |
| Anti-NMDAR | ND | Y | Y |

**Note to Table S1:** Y: yes. ND: not done

**Table S2:** Assignment of patient groups with confirmed CNS infections

| **Patient group** | **Evidence of microbial/antibody signature in CSF by** |
| --- | --- |
| Bacterial meningitis | Routine culture, and/or Gram stain and/or PCR |
| Tuberculous meningitis | Ziehl-Neelsen staining, and/or GenXpert and/or MGIT* |
| Encephalitis | PCR and/or IgM |
| Cryptococcal meningitis | India Ink Stain and/or Cryptococcal FLA and/or culture |
| Eosinophilic meningitis | PCR |
| Anti-NMDAR encephalitis | IgG against NMDA receptor |

**Note to Table S2:** *Mycobacteria Growth Indicator tubes

**Table S3. Baseline characteristics of the discovery and validation cohort**

|  | **Discovery cohort** | | | | **Validation cohort** | | | | | | |
| --- | --- | --- | --- | --- | --- | --- | --- | --- | --- | --- | --- |
|  | TBM (N=20) | Encephalitis (N=10) | BM^♪^ (N=10) | Non-CNS infections (N=5) | BM (N=64)^#^ | TBM (N=122)^♫^ | Encephalitis (N=92)^$^ | Anti-NMDAR (N=17) | Eosinophilic meningitis (N=10) | Cryptococcal meningitis (N=14) | Non-CNS infections (N=43) |
| **Demographics** |  |  |  |  |  |  |  |  |  |  |  |
| Age in years | 40(23-75) | 33(18-53) | 49(23-74) | 58(0-70) | 55 (17-87) | 41(17-87) | 31 (16-78) | 25(17-48) | 30(18-60) | 35.5(22-68) | 48(20-92) |
| Gender | 11/9 | 8/2 | 7/3 | 2/3 | 44/20 | 97/25 | 54/38 | 9/8 | 4/6 | 10/4 | 25/18 |
| Ho Chi Minh City origin | 5(25) | 2(20) | 4(44.4) | 2(40) | 12(18.8) | 29(23.8) | 27(29.3) | 3(17.6) | 2(20) | 4(28.6) | 10(23.3) |
| **Illness day at enrollment** | 14(0-60) | 5(0-10) | 2(0-13) | 4(3-18) | 4 (1-30) | 12 (2-90) | 6(1-90) | 22(6-37) | 25.5(7-60) | 18(4-30) | 5(1-60) |
| **Length of hospital stay** | 24(0-59) | 5(0-67) | 18(13-26) | 2(1-17) | 14 (1-119) | 26(0-162) | 11(0-118) | 41(27-102) | 11(1-23) | 23(0-134) | 8(0-75) |
| **Clinical signs/symptoms** |  |  |  |  |  |  |  |  |  |  |  |
| Fever (n,%) | 19(95) | 10(100) | NA | 5(100) | 59(96.7) | 117(96.7) | 83(92.2) | 13(76.5) | 7(70) | 12(85.7) | 36(87.8) |
| Headache (n,%) | 18(90) | 10(100) | 6(66.7) | 3(60) | 57(91.9) | 113(96.6) | 64(76.2) | 9(64.3) | 10(100) | 13(92.9) | 15(40.5) |
| Cranial nerve palsy (n,%) | 4(20) | 2(20) | NA | 0 | 5(7.8) | 23(18.9) | 9(9.8) | 0 | 3(30) | 4(28.6) | 3(7) |
| Hemiplegia (n,%) | 1(5) | 2(20) | NA | 0 | 1(1.6) | 12(9.8) | 4(4.3) | 0 | 1(10) | 1(7.1) | 3(7) |
| Paraplegia (n,%) | 0 | 0 | NA | 0 | 1(1.6) | 10(8.2) | 3(3.3) | 0 | 1(10) | 1(7.1) | 4(9.3) |
| Tetraplegia (n,%) | 0 | 0 | NA | 0 | 1(1.6) | 5(4.1) | 3(3.3) | 0 | 0 | 1(7.1) | 4(9.3) |
| Convulsions (n,%) | 1(5) | 1(10) | NA | 0 | 1 (1.6) | 2(1.7) | 15(16.5) | 0 | 0 | 0 | 5(11.9) |
| Neck stiffness (n,%) | 17(85) | 9(90) | NA | 4(80) | 47 (77) | 57(47.6) | 30(33.7) | 5(29.4) | 5(50) | 7(50) | 8(19.5) |
| GCS** at enrolment (median, range) | 14(8-15) | 11(7-15) | NA | 14(10-15) | 12 (3-15) | 14(4-15) | 11(3-15) | 11(6-14) | 15(9-15) | 13(8-15) | 13(7-15) |
| HIV positive, (n%) | 2(20) | 1(10) | NA | 0 | 0 | 22(24.2) | 0 | 0 |  |  | 1(2.3) |
| **CSF examinations** |  |  |  |  |  |  |  |  |  |  |  |
| CSF leukocyte count (per mm3) | 317 (58-896) | 209 (18-1571) | 11000 (500-19200) | 5(1-93) | 1924 (24-51810) | 312(3-3969) | 43(1-909) | 23(6-187) | 501(140-1101) | 36.5(2-357) | 2(1-2700) |
| CSF neutrophils (%) | 26(3-93) | 3(0-18) | 91.5(83-98) | 20(0-87) | 83 (10-98) | 26(0-95) | 14(0-91) | 14(9-65) | 12(7-42) | 26.5(0-67) | 30(0-93) |
| CSF lymphocytes (%) | 74(7-94) | 96(0-98) | 6(0.9-17) | 13(0-99) | 17 (2-90) | 73(5-92) | 84(0-94) | 86(35-91) | 47(20-70) | 67.5(33-86) | 50(0-99) |
| CSF/blood glucose ratio | 0.19  (0.04-0.4) | 0.61  (0.5-7.66) | NA | 0.65  (0.41-0.67) | 0.3 (0-1) | 0.3(0.1-0.7) | 4(1.7-7.7) | 0.8(0.5-1.4) | 0.4(0.4-0.9) | 0.3(0-0.6) | 0.7(0.4-1.3) |
| CSF lactate (mmol/L) | 6.77  (3.21-12.43) | 2.79  (1.88-3.52) | NA | 3.65  (1.78-7.3) | 9.9 (2.3-28.2) | 5(1.9-12.8) | 2.5(1.3-6.6) | 1.9(1.4-2.7) | 2.7(2.2-4.2) | 4.9(2.7-12.7) | 2.5(1.4-6.4) |
| Total protein (g/L) | 2(1.1-4.1) | 0.8(0.3-2.4) | NA | 0.5(0.3-2.6) | 2.3 (0.3-8.7) | 1.9(0.2-29.8) | 0.7(0.1-3.2 | 0.3(0.2-0.8) | 0.8(0.2-3.9) | 0.6(0.4-1.8) | 0.4(0.2-3.2) |
| **Discharge mRS^** |  |  |  | . |  |  |  |  |  |  |  |
| 0 | 3(15) | 0 | NA | 1(20) | 8(12.5) | 22(18) | 17(18.2) | 0 | 0 | 0 | 5(11.6) |
| 1 | 3(15) | 2(20) | NA | 0 | 6(9.4) | 16(13.1) | 19(20.7) | 2(11.8) | 3(30) | 0 | 6(14) |
| 2 | 5(25) | 0 | NA | 2(40) | 12(18.8) | 15(13.2) | 19(20.7) | 0 | 4(40) | 1(7.1) | 8(18.6) |
| 3 | 4(20) | 4(40) | NA | 1(20) | 20(31.3) | 14(11.5) | 12(13) | 6(35.3) | 1(10) | 4(28.6) | 8(18.6) |
| 4 | 1(5) | 2(20) | NA | 0 | 11(17.2) | 22(18) | 13(14.1) | 3(17.6) | 1(10) | 3(21.4) | 9(20.9) |
| 5 | 2(10) | 2(20) | NA | 0 | 3(4.7) | 17(13.7) | 10(10.9) | 5(29.4) | 1(10) | 1(7.1) | 6(14) |
| 6 | 2(10) | 0 | NA | 1(20) | 4(6.3) | 16(13.1) | 2(2.2) | 1(5.9) | 0 | 5(35.7) | 1(2.3) |

**Note to Table S3:** ^♪^outcomes at discharge were recorded as full recovery (n=4) or neurological deficit (n=4)*****due to the small sample size, data on two cases with neurotoxoplamosis are not shown, ^#^including 44 laboratory confirmed cases, ^♫^including 95 laboratory confirmed cases, ^$^including 23 laboratory confirmed cases, **Glasgow coma score, ^Modified Rankin Scale (0: full recovery with no symptoms, 1: No significant disability, 2: Slight disability, 3: Moderate disability, 4: Moderately severe disability, 5: Severe disability, and 6: Dead); BM: bacterial meningitis, TBM: tuberculous meningitis; Data are number (%), continuous variables are presented as median (range)

**Table S4:** The frequency of clinical conditions among 43 patients without CNS infections

| **Syndrome** | **Number of cases (n)** |
| --- | --- |
| Alcoholic | 3 |
| Cerebral infarction | 4 |
| Cerebral tumor | 1 |
| Cirrhosis | 1 |
| Endocrine disorder | 1 |
| Epileptic | 7 |
| Hypertension | 1 |
| Myasthenia gravis | 1 |
| Opioid disorder | 1 |
| Pneumonia | 1 |
| Post malaria neurological syndrome | 1 |
| Post viral infection syndrome | 5 |
| Psychiatric disorder | 2 |
| Sepsis | 12 |
| Spleen abscess | 1 |
| Wilson | 1 |

**Table S5. List of marker candidates identified by mass spectrometry analysis**

| **BM group** | | | | | | | |
| --- | --- | --- | --- | --- | --- | --- | --- |
| **No** | **Protein ID** | **Protein name** | **Gene name** | **Mean intensity of BM (log_2_)** | **Mean intensity of Other (log_2_)** | **Difference in intensity between BM and Others** | **-Log (p value)** |
| 1 | P06744 | Glucose-6-phosphate isomerase | GPI | -19.99 | -24.54 | -4.55 | 7.74 |
| 2 | P60660-2 | Myosin light polypeptide 6 | MYL6 | -19.77 | -26.05 | -6.28 | 7.3 |
| 3 | P28676 | Grancalcin | GCA | -21.18 | -26.58 | -5.4 | 7.11 |
| 4 | P11413-2 | Glucose-6-phosphate 1-dehydrogenase | G6PD | -21.17 | -26.4 | -5.23 | 6.31 |
| 5 | P26583 | High mobility group protein B2 | HMGB2 | -20.83 | -26.23 | -5.41 | 5.3 |
| 6 | P05109 | Protein S100-A8 | S100A8 | -14.87 | -20.83 | -5.96 | 5.87 |
| 7 | P05164-2 | Myeloperoxidase | MPO | -18.03 | -23.71 | -5.68 | 5.84 |
| 8 | P06702 | Protein S100-A9 | S100A9 | -14.55 | -21.29 | -6.75 | 5.84 |
| 9 | P43490 | Nicotinamide phosphoribosyltransferase | NAMPT | -21.54 | -26.07 | -4.53 | 5.82 |
| 10 | P80188-2 | Neutrophil gelatinase-associated lipocalin | LCN2 | -17.82 | -23.6 | -5.78 | 5.81 |
| 11 | P22894 | Neutrophil collagenase | MMP8 | -19.34 | -24.07 | -4.75 | 5.77 |
| 12 | P50395 | Rab GDP dissociation inhibitor beta | GDI2 | -20.2 | -24.66 | -4.46 | 5.74 |
| 13 | P20160 | Azurocidin | AZU1 | -20.45 | -26.12 | -5.67 | 5.61 |
| 14 | P41218 | Myeloid cell nuclear differentiation antigen | MNDA | -19.89 | -24.52 | -4.63 | 5.60 |
| 15 | P61160 | Actin-related protein 2 | ACTR2 | -21.19 | -25.46 | -4.27 | 5.47 |
| 16 | O15144 | Actin-related protein 2/3 complex subunit 2 | ARPC2 | -19.86 | -25.15 | -5.29 | 5.47 |
| 17 | P08670 | Vimentin | VIM | -18.54 | -22.2 | -3.66 | 5.46 |
| 18 | P08107 | Heat shock 70 kDa protein 1A | HSPA1A | -19.45 | -24.1 | -4.65 | 5.37 |
| 19 | P30044-2 | Peroxiredoxin-5, mitochondrial | PRDX5 | -20.84 | -25.66 | -4.82 | 5.23 |
| 20 | P04040 | Catalase | CAT | -19.23 | -24.71 | -5.48 | 5.22 |
| 21 | P09429 | High mobility group protein B1 | HMGB1 | -21.35 | -25.89 | -4.53 | 5.12 |
| 22 | P61158 | Actin-related protein 3 | ACTR3 | -20.73 | -25.39 | -4.66 | 5.03 |
| 23 | P35579 | Myosin-9 | MYH9 | -21.18 | -26.72 | -5.54 | 5.02 |
| 24 | P04083 | Annexin A1 | ANXA1 | -19.86 | -25.38 | -5.52 | 4.81 |
| 25 | P49913 | Cathelicidin antimicrobial peptide | CAMP | -20.72 | -24.6 | -3.88 | 4.74 |
| 26 | P12814-3 | Alpha-actinin-1 | ACTN1 | -20.87 | -25.06 | -4.18 | 4.73 |
| 27 | U3KPS2 | Myeloblastin | PRTN3 | -19.02 | -22.98 | -3.96 | 4.71 |
| 28 | P01040 | Cystatin-A | CSTA | -18.79 | -24.16 | -5.37 | 4.7 |
| 29 | Q6UX06 | Olfactomedin-4 | OLFM4 | -22.21 | -26.55 | -4.33 | 4.69 |
| 30 | P52209-2 | 6-phosphogluconate dehydrogenase, decarboxylating | PGD | -19.4 | -24.03 | -4.63 | 4.67 |
| 31 | P37837 | Transaldolase | TALDO1 | -19.90 | -24.88 | -4.98 | 4.6 |
| 32 | P51149 | Ras-related protein Rab-7a | RAB7A | -21.76 | -25.96 | -4.21 | 4.59 |
| 33 | P08246 | Neutrophil elastase | ELANE | -17.89 | -23.13 | -5.24 | 4.59 |
| 34 | O15143 | Actin-related protein 2/3 complex subunit 1B | ARPC1B | -21.64 | -26.41 | -4.77 | 4.58 |
| 35 | O43707 | Alpha-actinin-4 | ACTN4 | -21.84 | -25.85 | -4.01 | 4.52 |
| 36 | P08311 | Cathepsin G | CTSG | -19.38 | -24.24 | -4.86 | 4.48 |
| 37 | P59998-3 | Actin-related protein 2/3 complex subunit 4 | ARPC4 | -19.92 | -24.16 | -4.24 | 4.39 |
| 38 | P61626 | Lysozyme C | LYZ | -16.34 | -18.05 | -1.71 | 4.39 |
| 39 | P30041 | Peroxiredoxin-6 | PRDX6 | -21.56 | -25.35 | -3.79 | 4.35 |
| 40 | P00338-3 | L-lactate dehydrogenase A chain | LDHA | -20.19 | -23.1 | -2.91 | 4.24 |
| 41 | Q05315 | Galectin-10 | CLC | -21.36 | -25.34 | -3.98 | 4.18 |
| 42 | P09960 | Leukotriene A-4 hydrolase | LTA4H | -21.47 | -24.95 | -3.48 | 4.15 |
| 43 | O14950 | Myosin regulatory light chain 12B | MYL12B | -21.26 | -25.66 | -4.4 | 4.12 |
| 44 | P09211 | Glutathione S-transferase P | GSTP1 | -18.82 | -23.38 | -4.56 | 4.1 |
| 45 | P00491 | Purine nucleoside phosphorylase | PNP | -21.14 | -25.57 | -4.43 | 4.07 |
| 46 | P18428 | Lipopolysaccharide-binding protein | LBP | -20.82 | -24.91 | -4.09 | 4.05 |
| 47 | P60709 | Actin, cytoplasmic 1 | ACTB | -16.18 | -17.82 | -1.65 | 4.02 |
| 48 | P21333-2 | Filamin-A | FLNA | -21.85 | -26.23 | -4.38 | 4.01 |
| 49 | Q9ULZ3-2 | Apoptosis-associated speck-like protein containing a CARD | PYCARD | -20.63 | -24.76 | -4.13 | 3.88 |
| 50 | P47756-2 | F-actin-capping protein subunit beta | CAPZB | -21.9 | -26.33 | -4.44 | 3.85 |
| 51 | P62491-2 | Ras-related protein Rab-11A | RAB11A | -21.91 | -26.14 | -4.23 | 3.82 |
| 52 | Q01518 | Adenylyl cyclase-associated protein 1 | CAP1 | -20.93 | -25.4 | -4.47 | 3.77 |
| 53 | O15145 | Actin-related protein 2/3 complex subunit 3 | ARPC3 | -21.08 | -24.75 | -3.67 | 3.77 |
| 54 | O00299 | Chloride intracellular channel protein 1 | CLIC1 | -21.69 | -26.07 | -4.38 | 3.75 |
| 55 | P35754 | Glutaredoxin-1 | GLRX | -20.49 | -24.84 | -4.36 | 3.62 |
| 56 | E9PR52 | Chitinase-3-like protein 2 | CHI3L2 | -21.29 | -25.72 | -4.43 | 3.6 |
| 57 | P02788-2 | Lactotransferrin | LTF | -18.8 | -24.04 | -5.24 | 3.38 |
| 58 | P18206-2 | Vinculin | VCL | -22.71 | -26.28 | -3.58 | 3.34 |
| 59 | P52566 | Rho GDP-dissociation inhibitor 2 | ARHGDIB | -18.87 | -22.34 | -3.47 | 3.31 |
| 60 | P62942 | Peptidyl-prolyl cis-trans isomerase FKBP1A | FKBP1A | -19.83 | -24.1 | -4.27 | 3.27 |
| TBM group | | | | | | | |
| **No** | **Protein ID** | **Protein name** | **Gene name** | **Mean intensity of BM (log_2_)** | **Mean intensity of Other (log_2_)** | **Difference in intensity between TBM and Others** | **-Log (p value)** |
| 1 | P25311 | Zinc-alpha-2-glycoprotein | AZGP1 | -16.21 | -17.29 | -1.08 | 9.22 |
| 2 | P23381 | Tryptophan-tRNA ligase | WARS | -20.06 | -23.81 | -3.75 | 5.58 |
| 3 | P29622 | Kallistatin | SERPINA4 | -20.65 | -22.77 | -2.13 | 4.39 |
| 4 | P02746 | Complement C1q subcomponent subunit B | C1QB | -18.32 | -19.31 | -0.99 | 4.35 |
| 5 | A0A075B6J0 | Immunoglobulin lambda variable 1-40 | IGLV1-40 | -17.36 | -20.25 | -2.9 | 4.25 |
| 6 | P02749 | Beta-2-glycoprotein 1 | APOH | -18.32 | -19.33 | -1.01 | 4.13 |
| 7 | P32455 | Guanylate-binding protein 1 | GBP1 | -23.23 | -25.44 | -2.21 | 3.41 |
| 8 | P16070-10 | CD44 antigen | CD44 | -22.26 | -23.46 | -1.19 | 3.23 |
| 9 | P02747 | Complement C1q subcomponent subunit C | C1QC | -17.8 | -18.93 | -1.13 | 3.14 |
| 10 | P01591 | Immunoglobulin J chain | JCHAIN | -19.12 | -22.1 | -2.98 | 2.93 |
| 11 | Q8WVN6 | Secreted and transmembrane protein 1 | SECTM1 | -20.96 | -23.77 | -2.81 | 2.88 |
| 12 | Q96IY4 | Carboxypeptidase B2 | CPB2 | -21.79 | -24.05 | -2.26 | 2.83 |
| 13 | Q14624 | Inter-alpha-trypsin inhibitor heavy chain H4 | ITIH4 | -22.42 | -24.14 | -1.72 | 2.82 |
| 14 | P01625 | Immunoglobulin kappa variable 4-1 | IGKV4-1 | -23.58 | -26.87 | -3.29 | 2.78 |
| 15 | O15204 | ADAM DEC1 | ADAMDEC1 | -22.75 | -25.26 | -2.51 | 2.64 |
| 16 | P19971-2 | Thymidine phosphorylase | TYMP | -22.78 | -25.01 | -2.24 | 2.57 |
| 17 | A0A075B6J9 | Immunoglobulin lambda variable 2-18 | IGLV2-18 | -21.35 | -24.22 | -2.87 | 2.37 |
| 18 | P01596 | Immunoglobulin kappa variable 1-5 | IGKV1-5 | -21.02 | -23.7 | -2.69 | 2.36 |
| 19 | P02743 | Serum amyloid P-component | APCS | -23.62 | -25.81 | -2.19 | 2.3 |

**Table S6**. **Results of analysis comparing the diagnostic value of LCN2 in distinguishing between patients with confirmed and clinically suspected bacterial meningitis**

| **cBM vs. sBM** | **Cut-off** | **AUC**  **(95% CI)** | **Sensitivity**  **(95% CI)** | **Specificity**  **(95% CI)** | **DOR**  **(95% CI)** |
| --- | --- | --- | --- | --- | --- |
| LCN2 (ng/ml) | 452.3 | 0.74  (0.6-0.88) | 0.82  (0.68-0.9) | 0.6  (0.39-0.78) | 6.8  (2.1-21.9) |
| CSF leukocytes (cell per mm^3^) | 1533 | 0.61  (0.47-0.76) | 0.61  (0.47-0.74) | 0.65  (0.43-0.82) | 3.0  (1-8.9) |
| CSF lactate (mmol/L) | 9.0 | 0.84  (0.74-0.95) | 0.77  (0.63-0.87) | 0.8  (0.58-0.92) | 13.6  (3.7-50.1) |
| CSF/blood glucose ratio | 0.2 | 0.76  (0.63-0.88) | 0.57  (0.42-0.7) | 0.95  (0.76-0.99) | 25  (3.1-203.6) |
| CSF protein (g/L) | 2.1 | 0.74  (0.61-0.86) | 0.66  (0.51-0.78) | 0.75  (0.53-0.89) | 5.8  (1.8-19) |
| CSF White cell count+lactate+CSF/blood glucose level+CSF protein | NA | 0.87  (0.77-0.97) | 0.95  (0.85-0.99) | 0.65  (0.43-0.82) | 39  (7.2-21.4) |
| CSF White cell count+lactate+CSF/blood glucose level+CSF protein+lipocalin 2 | NA | 0.87  (0.77-0.96) | 0.95  (0.85-0.99) | 0.65  (0.43-0.82) | 39  (7.2-21.4) |


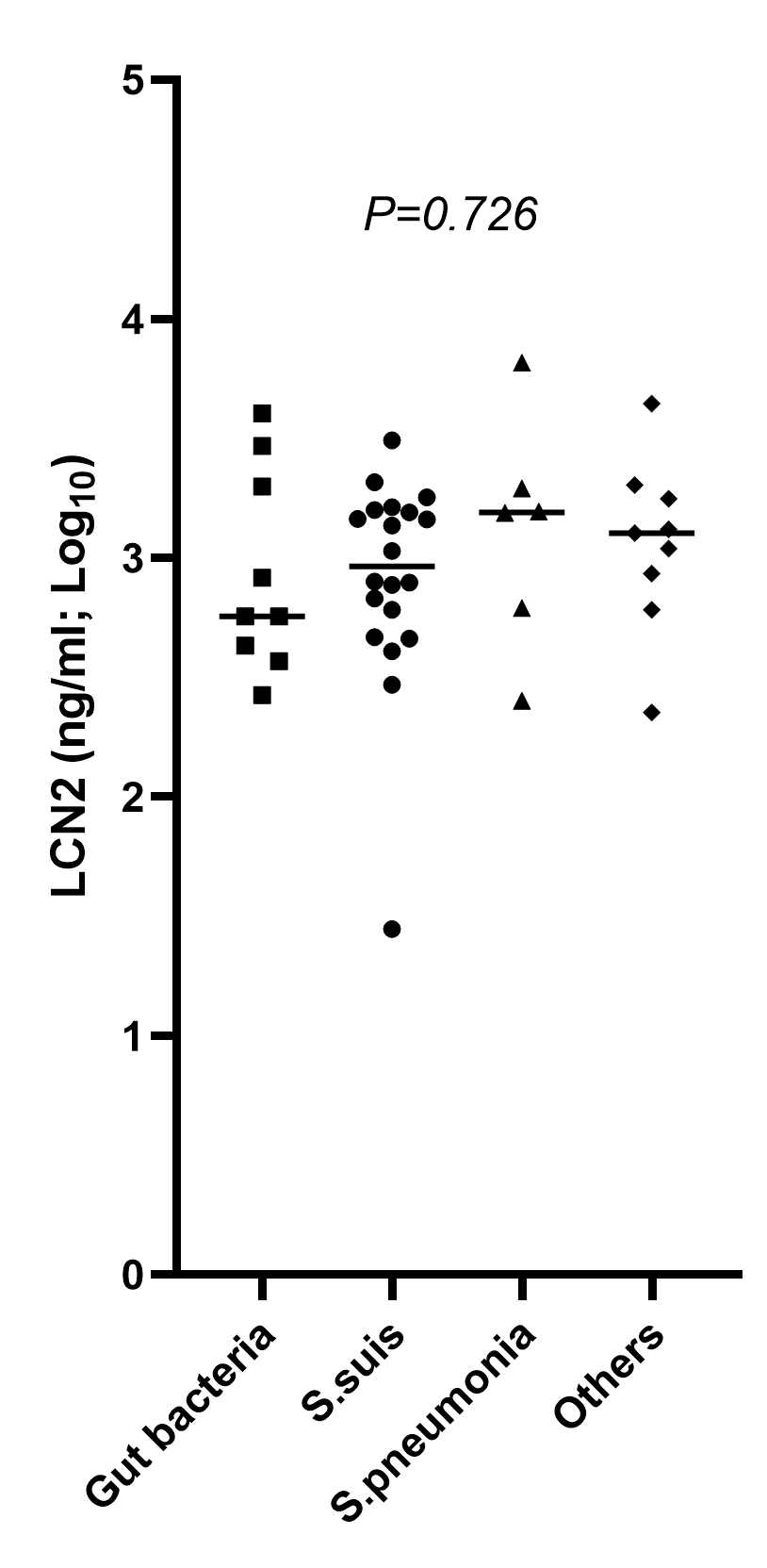
**
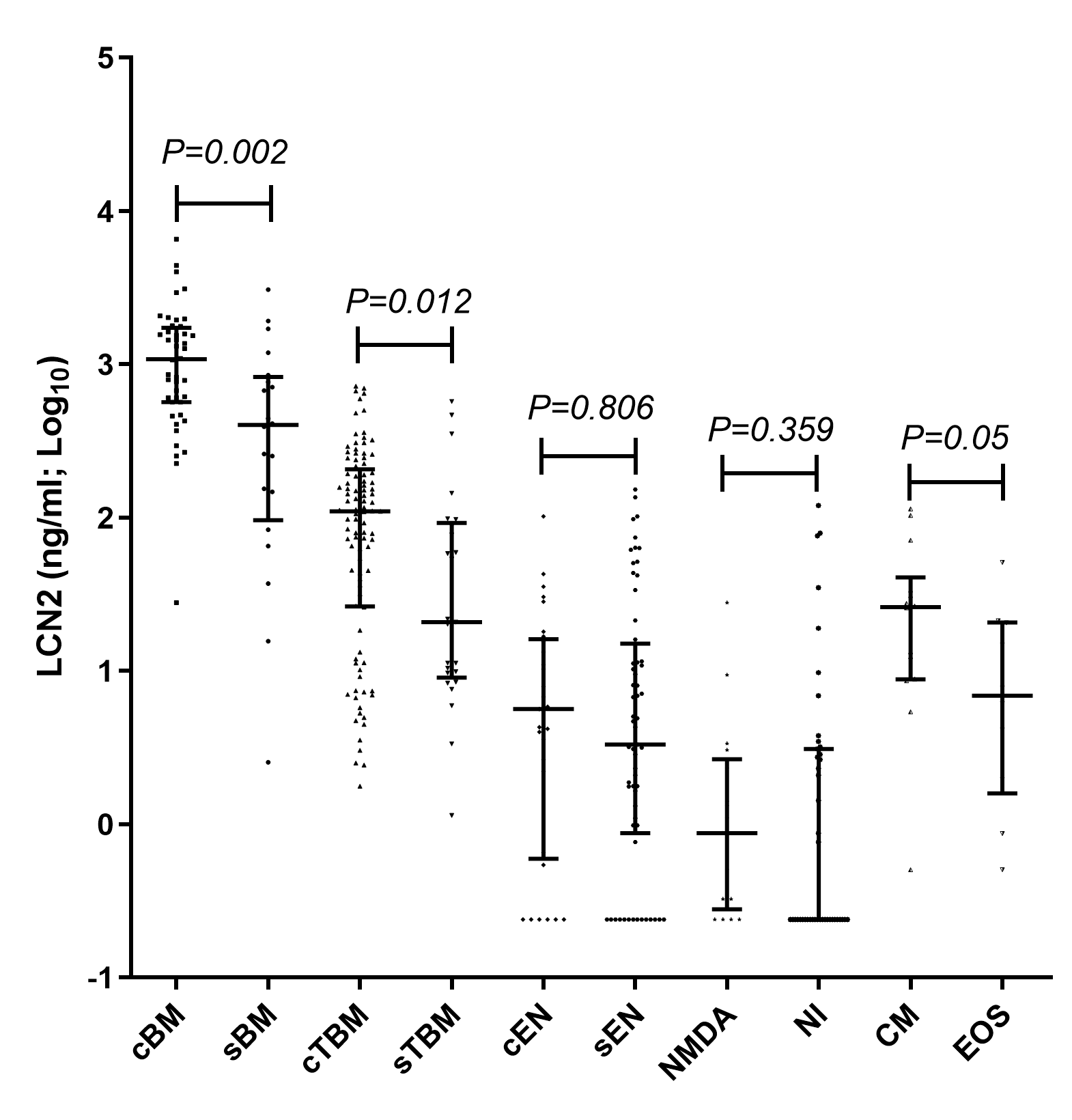
**

**Figure S1. Plots showing the distribution of LCN2 concentrations in various patients; A) patients with laboratory confirmed or clinically suspected CNS infections and non-CNS infections, B) confirmed bacterial meningitis patients infected with**

**Note to Figure S1:** cBM: confirmed bacterial meningitis, sBM: clinically suspected bacterial meningitis, cTBM: confirmed tuberculous meningitis, sTBM: clinically suspected tuberculous meningitis, cEN: confirmed encephalitis, sEN: clinically suspected encephalitis, NMDA: anti-NDMAR encephalitis, NI: non-CNS infections, CM: crytococcal meningitis, EOS: eosinophilic meningitis; Gut bacteria: *E. coli*, *E. faecalis*, *E. gallinarum*, *S. agalactiae* , and *S. gallolyticus,* Others: *N. meningitidis*, *B. pseudomallei*, *S. aureus*, and gram staining positive only patients.


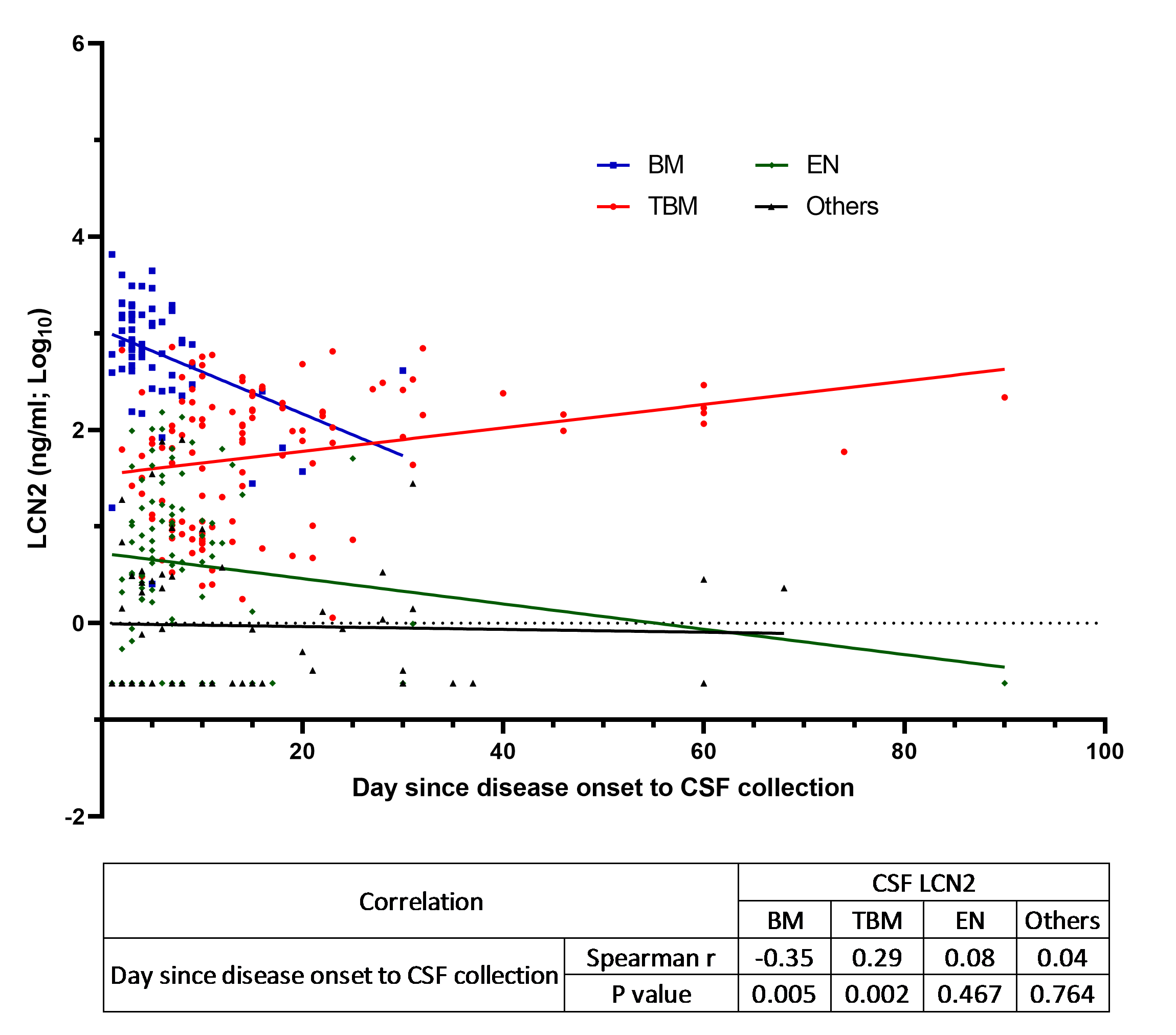


**Figure S2**: Association between CSF LCN2 levels and illness day at enrollment among different patient groups


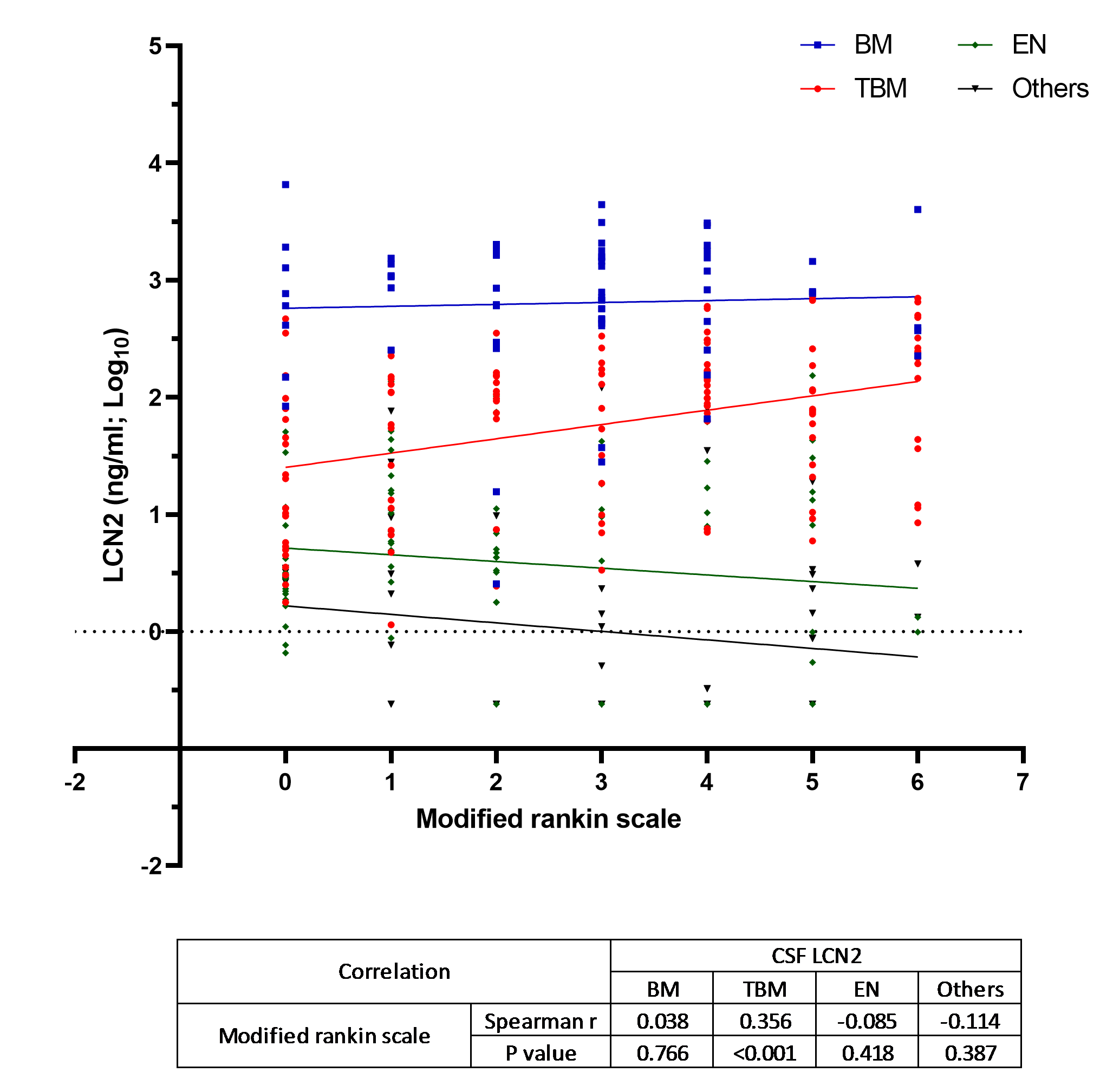


**Figure S3**: Association between CSF LCN2 levels and in-hospital outcomes

| **cBM prediction** | **Cut-off**  **values** | **AUC**  **(95% CI)** | **Sensitivity**  **(95% CI)** | **Specificity**  **(95% CI)** | **DOR**  **(95% CI)** |
| --- | --- | --- | --- | --- | --- |
| Lipocalin 2 (ng/ml) | 221.3 | 0.98  (0.96-1) | 0.98  (0.88-1) | 0.91  (0.88-0.94) | 453.2  (59.9-3426.3) |
| CSF leukocytes (cell per mm^3^) | 427 | 0.91  (0.86-0.96) | 0.86  (0.73-0.94) | 0.8  (0.75-0.84) | 25.3  (10.2-62.7) |
| CSF lactate (mmol/L) | 5.8 | 0.95  (0.91-0.98) | 0.91  (0.79-0.96) | 0.84  (0.79-0.88) | 52.5  (17.9-153.5) |
| CSF/blood glucose ratio | <0.2 | 0.78  (0.69-0.86) | 0.57  (0.42-0.7) | 0.92  (0.88-0.94) | 14.3  (6.9-29.4) |
| CSF protein (g/L) | 2.9 | 0.81  (0.75-0.88) | 0.57  (0.42-0.7) | 0.91  (0.87-0.94) | 13.3  (6.5-27.2) |
| CSF white cell count+lactate+CSF/blood glucose level+CSF protein | NA | 0.97  (0.95-1) | 0.93  (0.82-0.98) | 0.92  (0.88-0.94) | 148.2  (42.8-512.8) |
| CSF white cell count+lactate+CSF/blood glucose level+CSF protein+lipocalin 2 | NA | 0.99  (0.98-1) | 0.93  (0.82-0.98) | 0.97  (0.94-0.98) | 435.8  (113.3-1676.1) |


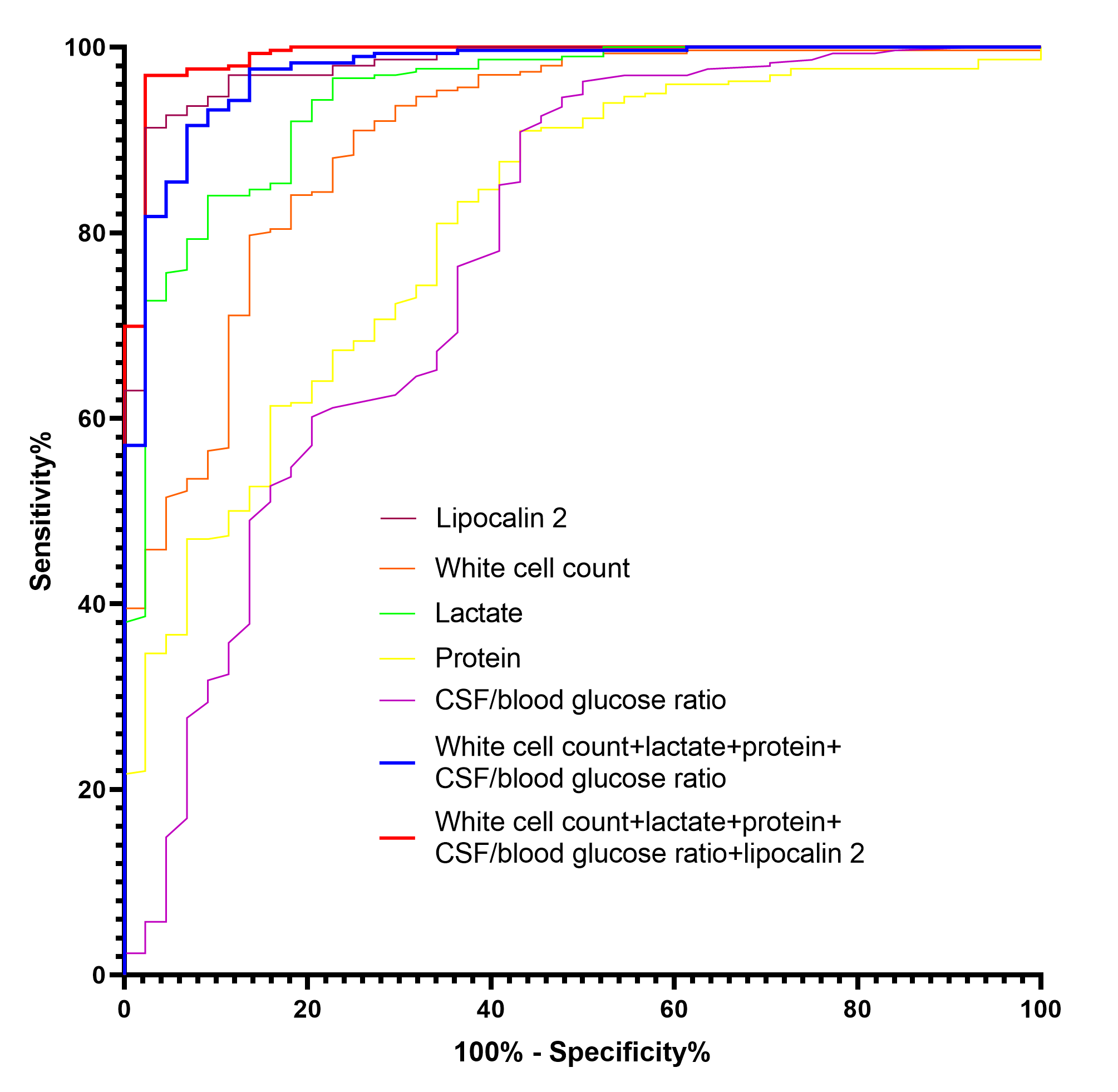


**A**

**A**


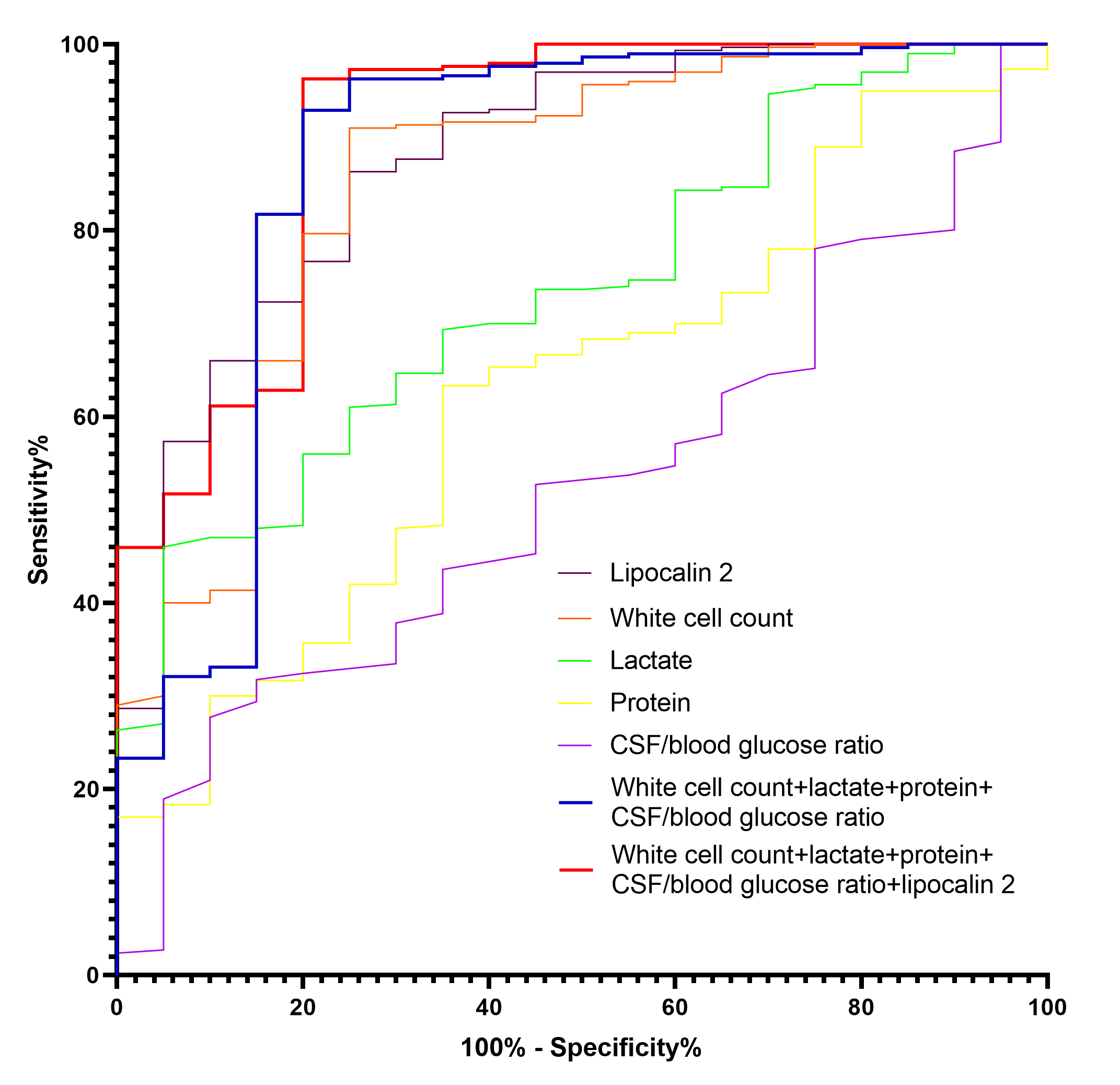


**B**

**B**

| **sBM prediction** | **Cut-off**  **values** | **AUC**  **(95% CI)** | **Sensitivity**  **(95% CI)** | **Specificity**  **95% CI)** | **DOR**  **(95% CI)** |
| --- | --- | --- | --- | --- | --- |
| Lipocalin 2 (ng/ml) | 146.5 | 0.88  (0.79-0.96) | 0.75  (0.53-0.89) | 0.86  (0.82-0.9) | 18.9  (6.5-54.9) |
| CSF white cell count (per mm^3^) | 709 | 0.85  (0.75-0.95) | 0.75  (0.53-0.89) | 0.91  (0.87-0.94) | 30.3  (10.2-89.9) |
| CSF lactate (mmol/L) | 3.0 | 0.73  (0.64-0.83) | 0.95  (0.76-0.99) | 0.46  (0.41-0.52) | 16.2  (2.1-122.5) |
| CSF/blood glucose ratio | <0.7 | 0.52  (0.41-0.64) | 0.9  (0.7-0.97) | 0.28  (0.24-0.34) | 3.6  (0.8-15.7) |
| CSF protein (g/L) | 1.2 | 0.62  (0.51-0.74) | 0.65  (0.43-0.82) | 0.63  (0.58-0.69) | 3.2  (1.2-8.3) |
| CSF white cell count+lactate+CSF/blood glucose level+CSF protein | NA | 0.87  (0.76-0.98) | 0.8  (0.58-0.92) | 0.93  (0.89-0.95) | 52.4  (16.1-170.8) |
| CSF white cell count+lactate+CSF/blood glucose level+CSF protein+lipocalin 2 | NA | 0.9  (0.83-0.98) | 0.8  (0.58-0.92) | 0.96  (0.94-0.98) | 103.6  (29.7-361.8) |

**Figure S4. Diagnostic performance of LCN2 in discriminating between laboratory confirmed (A) or clinically suspected bacterial meningitis patients (B) and other clinical entities and in comparison with existing biomarkers.**

**Note to Figure S4:** NA: not applicable
